# The impact of air pollution on deaths, disease burden, and life expectancy across the states of India: the Global Burden of Disease Study 2017

**DOI:** 10.1016/S2542-5196(18)30261-4

**Published:** 2019-01

**Authors:** India State-Level Disease Burden Initiative Air Pollution Collaborators, Kalpana Balakrishnan, Sagnik Dey, Tarun Gupta, R S Dhaliwal, Michael Brauer, Aaron J Cohen, Jeffrey D Stanaway, Gufran Beig, Tushar K Joshi, Ashutosh N Aggarwal, Yogesh Sabde, Harsiddha Sadhu, Joseph Frostad, Kate Causey, William Godwin, D K Shukla, G Anil Kumar, Chris M Varghese, Pallavi Muraleedharan, Anurag Agrawal, R M Anjana, Anil Bhansali, Deeksha Bhardwaj, Katrin Burkart, Kelly Cercy, Joy K Chakma, Sourangsu Chowdhury, D J Christopher, Eliza Dutta, Melissa Furtado, Santu Ghosh, Aloke G Ghoshal, Scott D Glenn, Randeep Guleria, Rajeev Gupta, Panniyammakal Jeemon, Rajni Kant, Surya Kant, Tanvir Kaur, Parvaiz A Koul, Varsha Krish, Bhargav Krishna, Samantha L Larson, Kishore Madhipatla, P A Mahesh, Viswanathan Mohan, Satinath Mukhopadhyay, Parul Mutreja, Nitish Naik, Sanjeev Nair, Grant Nguyen, Christopher M Odell, Jeyaraj D Pandian, Dorairaj Prabhakaran, Poornima Prabhakaran, Ambuj Roy, Sundeep Salvi, Sankar Sambandam, Deepika Saraf, Meenakshi Sharma, Aakash Shrivastava, Virendra Singh, Nikhil Tandon, Nihal J Thomas, Anna Torre, Denis Xavier, Geetika Yadav, Sujeet Singh, Chander Shekhar, Theo Vos, Rakhi Dandona, K Srinath Reddy, Stephen S Lim, Christopher J L Murray, S Venkatesh, Lalit Dandona

## Abstract

**Background:**

Air pollution is a major planetary health risk, with India estimated to have some of the worst levels globally. To inform action at subnational levels in India, we estimated the exposure to air pollution and its impact on deaths, disease burden, and life expectancy in every state of India in 2017.

**Methods:**

We estimated exposure to air pollution, including ambient particulate matter pollution, defined as the annual average gridded concentration of PM_2.5_, and household air pollution, defined as percentage of households using solid cooking fuels and the corresponding exposure to PM_2.5_, across the states of India using accessible data from multiple sources as part of the Global Burden of Diseases, Injuries, and Risk Factors Study (GBD) 2017. The states were categorised into three Socio-demographic Index (SDI) levels as calculated by GBD 2017 on the basis of lag-distributed per-capita income, mean education in people aged 15 years or older, and total fertility rate in people younger than 25 years. We estimated deaths and disability-adjusted life-years (DALYs) attributable to air pollution exposure, on the basis of exposure–response relationships from the published literature, as assessed in GBD 2017; the proportion of total global air pollution DALYs in India; and what the life expectancy would have been in each state of India if air pollution levels had been less than the minimum level causing health loss.

**Findings:**

The annual population-weighted mean exposure to ambient particulate matter PM_2·5_ in India was 89·9 μg/m^3^ (95% uncertainty interval [UI] 67·0–112·0) in 2017. Most states, and 76·8% of the population of India, were exposed to annual population-weighted mean PM_2·5_ greater than 40 μg/m^3^, which is the limit recommended by the National Ambient Air Quality Standards in India. Delhi had the highest annual population-weighted mean PM_2·5_ in 2017, followed by Uttar Pradesh, Bihar, and Haryana in north India, all with mean values greater than 125 μg/m^3^. The proportion of population using solid fuels in India was 55·5% (54·8–56·2) in 2017, which exceeded 75% in the low SDI states of Bihar, Jharkhand, and Odisha. 1·24 million (1·09–1·39) deaths in India in 2017, which were 12·5% of the total deaths, were attributable to air pollution, including 0·67 million (0·55–0·79) from ambient particulate matter pollution and 0·48 million (0·39–0·58) from household air pollution. Of these deaths attributable to air pollution, 51·4% were in people younger than 70 years. India contributed 18·1% of the global population but had 26·2% of the global air pollution DALYs in 2017. The ambient particulate matter pollution DALY rate was highest in the north Indian states of Uttar Pradesh, Haryana, Delhi, Punjab, and Rajasthan, spread across the three SDI state groups, and the household air pollution DALY rate was highest in the low SDI states of Chhattisgarh, Rajasthan, Madhya Pradesh, and Assam in north and northeast India. We estimated that if the air pollution level in India were less than the minimum causing health loss, the average life expectancy in 2017 would have been higher by 1·7 years (1·6–1·9), with this increase exceeding 2 years in the north Indian states of Rajasthan, Uttar Pradesh, and Haryana.

**Interpretation:**

India has disproportionately high mortality and disease burden due to air pollution. This burden is generally highest in the low SDI states of north India. Reducing the substantial avoidable deaths and disease burden from this major environmental risk is dependent on rapid deployment of effective multisectoral policies throughout India that are commensurate with the magnitude of air pollution in each state.

**Funding:**

Bill & Melinda Gates Foundation; and Indian Council of Medical Research, Department of Health Research, Ministry of Health and Family Welfare, Government of India.

## Introduction

Air pollution contributes substantially to premature mortality and disease burden globally, with a greater impact in low-income and middle-income countries than in high-income countries.^1^, ^2^ India has one of the highest exposure levels to air pollution globally.^1^ The major components of air pollution are ambient particulate matter pollution, household air pollution, and to a smaller extent ozone in the troposphere, the lowest layer of atmosphere. In India, the major sources of ambient particulate matter pollution are coal burning for thermal power production, industry emissions, construction activity and brick kilns, transport vehicles, road dust, residential and commercial biomass burning, waste burning, agricultural stubble burning, and diesel generators.^3^, ^4^, ^5^, ^6^, ^7^, ^8^, ^9^, ^10^, ^11^ Household air pollution is caused mainly by the residential burning of solid fuels for cooking and to some extent heating, the major types of which are wood, dung, agricultural residues, coal, and charcoal.^12^, ^13^, ^14^ Ground level ambient ozone is produced when nitrogen oxides and volatile organic compounds emitted from transport vehicles, power plants, factories, and other sources react in the presence of sunlight.^15^ Rapidly developing countries such as India face the dual challenge of exposures from both ambient and household air pollution.^16^ There has been an increasing focus on addressing air pollution in India by the government and other stakeholders in recent times.^17^, ^18^, ^19^, ^20^, ^21^, ^22^, ^23^, ^24^

**Research in context**

**Evidence before this study**

Existing evidence suggests that India, with a population of 1·38 billion people living across states at different levels of economic, social, and health development, has one of the highest air pollution levels in the world. Evidence also suggests that air pollution is a major risk factor for disease burden. We searched PubMed and publicly available reports for estimates of the burden attributable to air pollution, including ambient air pollution and household air pollution, across the states of India using the search terms “air pollutants”, “air pollution”, “ambient particulate matter pollution”, “burden”, “DALY”, “death”, “epidemiology”, "household air pollution", “impact”, “India”, “indoor pollution”, “life expectancy”, “morbidity”, “mortality”, “ozone concentration”, “PM2·5 exposure”, and “sources of emission” on Sept 14, 2018, without language or publication date restrictions. We found several previous studies that have estimated subnational variations in ambient particulate matter and household air pollution exposure in India and their contribution to deaths from various causes. However, a comprehensive understanding of the variations between the states of India in the exposure to the major components of air pollution, the associated deaths and disease burden, and the impact on life expectancy is not available in a single standardised framework to inform relevant policy interventions commensurate with the situation in each state.

**Added value of this study**

This study provides a comprehensive assessment of the exposure to air pollution and its impact on deaths, disease burden, and life expectancy in every state of India in 2017 using the unified Global Burden of Diseases, Injuries, and Risk Factors Study framework, which includes 359 diseases or injuries and 84 risk factors. Using improved GBD 2017 methods for air pollution, we report the separate impact of ambient particulate matter pollution and household air pollution for every state, avoiding overestimation of this impact in people exposed to both. Our findings highlight that 77% of India's population was exposed to an annual population-weighted mean PM_2·5_ greater than 40 μg/m^3^ in 2017, which is the level recommended by the National Ambient Air Quality Standards in India, and none of the Indian states met the WHO-recommended criteria of ambient particulate matter air quality of less than 10 μg/m^3^. Even with substantial increasing provision of clean cooking fuels in India, more than half of India's population was exposed to household air pollution from solid cooking fuels in 2017. We report that one out of every eight deaths in India in 2017 could be attributed to air pollution. This study shows that India has a higher proportion of global health loss due to air pollution than its proportion of the global population. The findings of this study suggest that the impact of air pollution on deaths and life expectancy in India might be lower than previously estimated, but this impact is still quite substantial.

**Implications of all the available evidence**

The high level of air pollution in India is a major public health and development issue that has significant implications for planetary health. There are large variations between the states of India in exposure to ambient particulate matter pollution and household air pollution and the consequent health loss and deaths. Although control of air pollution is needed all over India, the heterogeneity between the states should be taken into account in designing policies and interventions consistent with the magnitude and sources of air pollution in each state. In addition to the existing interventions, concerted multisectoral efforts are needed related to power production, industry, transport, fuel use, urban planning, construction, and agriculture for controlling air pollution in India to mitigate its impact. Public and policy focus on the control of air pollution in India is increasing, which should be sustained to translate this positive trend into effective interventions.

India had a population of 1·38 billion in 2017 spread across 29 states and seven union territories, many of which are as large as some countries and are at varying levels of development, leading to a heterogeneous distribution of health risks and their impact.^25^ The India State-Level Disease Burden Initiative has reported the overall trends of diseases, injuries, and risk factors from 1990 to 2016 for every state of India as part of the Global Burden of Diseases, Injuries, and Risk Factors Study (GBD) 2016, and also detailed trends of some major non-communicable diseases and suicide.^25^, ^26^, ^27^, ^28^, ^29^, ^30^, ^31^ According to these findings, air pollution was the second largest risk factor contributing to disease burden in India after malnutrition in 2016, with an increasing trend in exposure to ambient particulate matter pollution and a decreasing trend in household air pollution.^25^, ^26^ Another study has used satellite-based ambient particulate matter estimates for 2001–10 to highlight variations in the exposure levels at the district level in India and its contribution to deaths from various causes.^32^ These investigators also projected a continuing increase in ambient particulate matter pollution in India in the foreseeable future.^33^ Two studies have previously estimated the impact of air pollution on life expectancy in India.^34^, ^35^

Using improved air pollution methods in GBD 2017, we provide detailed findings on the exposure to ambient particulate matter pollution and household air pollution, and their separate impacts on deaths, disease burden, and life expectancy in every state of India, as well as the impact of overall air pollution, to inform policy and interventions.

## Methods

### Overview

The analysis and findings of air pollution presented in this report were produced by the India State-Level Disease Burden Initiative as part of GBD 2017. The work of this Initiative has been approved by the Health Ministry Screening Committee at the Indian Council of Medical Research and the ethics committee of the Public Health Foundation of India. A comprehensive description of the metrics, data sources, and statistical modelling for GBD 2017 has been reported elsewhere.^25^, ^26^, ^36^ The GBD 2017 methods relevant for this paper are summarised here and described in detail in the appendix (pp 3–15).

### Estimation of exposure to air pollution

The measure of exposure to ambient particulate matter pollution was the annual average PM_2·5_ concentration in the air at a spatial resolution of a 0·1° × 0·1° grid cell over the globe, which is 11 × 11 km at the equator.^36^, ^37^, ^38^, ^39^ The estimates of ambient PM_2·5_ exposure in India were based on multiple satellite-based aerosol optical depth data combined with a chemical transport model, and calibration of these with PM_2·5_ data from ground-level monitoring stations.^37^, ^38^, ^39^ The data inputs are listed in the appendix (pp 21–26). In cases in which data on average PM_10_ concentration were available but data on PM_2·5_ were not, estimates of ratios between the two were used to derive PM_2·5_ concentrations.^36^, ^39^ A description of the modelling approach used to arrive at the annual population-weighted mean PM_2·5_ estimates from a combination of satellite-based and ground-level data is published elsewhere.^37^, ^38^, ^39^ Estimates in GBD 2017 included a substantially increased number of ground measurements compared with previous GBD cycles, including 185 sites with PM_2·5_ measurements and 184 sites with PM_10_ measurements in India, and the model to calibrate satellite-based estimates to these measurements varied smoothly over space and time in regions with many measurements. Additionally, estimates of PM_2·5_ exposure uncertainty incorporate the posterior distribution in each grid cell from the calibration model. The methods for ambient particulate matter pollution estimation are provided in the appendix (pp 4–11).

The measure of household air pollution was exposure to PM_2·5_ due to use of solid cooking fuels (wood, dung, agricultural residues, coal, and charcoal), which was derived from the proportion of population using these fuels. Estimates of the proportion of population exposed to household air pollution from solid fuel use were modelled using spatiotemporal regression and Gaussian process regression techniques on population-based data on households using solid fuels. The average PM_2·5_ exposures from solid fuel use for different household members were derived from studies measuring 24-h kitchen and living area PM_2·5_ concentrations in households, estimating these for men, women, and children.^36^ The concentration of ambient PM_2·5_ for each location-year was then subtracted from these exposure estimates to provide an estimate of the incremental exposure due to household solid fuel use for cooking. This approach resulted in independent estimates for PM_2·5_ exposure due to ambient particulate matter and household solid fuel use. The major data sources for solid fuel use in India included the national health surveys such as the National Family Health Survey and the District Level Household Survey, nationwide surveys of the National Sample Survey Organisation, and the Census of India as well as many other published and unpublished epidemiological studies (appendix pp 21–26). The methods for household air pollution estimation are described elsewhere and a summary is provided in the appendix (pp 11–13).^36^

Ozone exposure was defined as the highest seasonal (6-month) mean daily maximum 8-h average concentrations of ozone in air as parts per billion for each 0·1° × 0·1° grid cell over the globe. These exposure estimates in GBD 2017 incorporated a new comprehensive global ozone ground measurement database.^40^ The burden attributable to ambient ozone pollution was estimated using chemical transport models. These methods are described elsewhere and in the appendix (pp 13, 14).^36^

### Estimation of deaths and disability-adjusted life-years (DALYs) attributable to air pollution

The GBD comparative risk assessment framework was used to estimate disease burden attributable to risk factors, as described elsewhere.^36^ The risk–outcome pairs were selected to comply with the World Cancer Research Fund classification grades of convincing or probable evidence for a biologically plausible association between exposure and disease outcomes reported in multiple epidemiological studies in different populations. These studies included prospective observational studies and randomised controlled trials. The relative risks for mortality from acute lower respiratory infections, ischaemic heart diseases, stroke, chronic obstructive pulmonary disease, lung cancer, and diabetes due to ambient and household exposure to PM_2·5_ were estimated using integrated exposure–response functions based on published relative risks at different PM_2·5_ concentrations, as described elsewhere and in the appendix (pp 4–13).^36^ The relative risk of cataract attributable to household use of solid fuels was generated from meta-analysis (appendix pp 11–13). The relative risk of chronic obstructive pulmonary disease attributable to ozone exposure was obtained from the literature (appendix pp 13, 14).

For each risk factor, the theoretical minimum risk exposure level was established as the lowest level of exposure below which its relationship with a disease outcome is not supported by the available evidence. The theoretical minimum risk exposure level for ambient particulate matter and household air pollution was defined as a population-weighted mean PM_2·5_ between 2·4 and 5·9 μg/m^3^, except for the attribution of cataract to household air pollution for which the theoretical minimum risk exposure level was defined as no exposure to solid fuel use for cooking.^36^ For ambient ozone pollution, the theoretical minimum risk exposure level was defined as a population-weighted concentration between 29·1 and 35·7 parts per billion. Relative risk estimates were based on the contrast between current exposure and the lowest theoretical minimum risk exposure level consistent with the available scientific evidence.

To differentiate the disease burden from PM_2·5_ exposure due to household solid fuel use and ambient particulate matter pollution, the attributable relative risk estimation approach using the integrated exposure–response function was modified in GBD 2017 compared with the previous GBD approach.^36^ Although everyone is exposed to some concentration of ambient particulate matter pollution, only a proportion of the population in each location use solid cooking fuels. For the proportion of the population not exposed to solid cooking fuel, the relative risk was based on the contrast between ambient PM_2·5_ concentration and its theoretical minimum risk exposure level. However, for the proportion of the population exposed to both household and ambient particulate matter pollution, a joint relative risk was calculated from the integrated exposure–response function according to the combined level of these exposures. This risk was divided between household air pollution and ambient particulate matter pollution on the basis of the proportion of each in the combined exposure. With this approach, the potential overestimation of disease burden among those individuals exposed to both household and ambient PM_2·5_ was avoided.

Population-attributable fractions for mortality and DALYs due to relative risks were estimated by location, year, age, and sex, using population attributable fractions derived from the published literature, as described in the appendix (pp 3–15) and elsewhere.^36^ GBD uses covariates, which are explanatory variables that have a known association with the outcome of interest, to arrive at the best possible estimate when data for the outcome are scarce but data for covariates are available.^36^, ^41^, ^42^ This approach was part of the estimation process for the findings presented in this report.

### Analysis presented in this paper

We report findings for 31 geographical units in India: the 29 states, the Union Territory of Delhi, and the union territories other than Delhi (combining the six smaller union territories of Andaman and Nicobar Islands, Chandigarh, Dadra and Nagar Haveli, Daman and Diu, Lakshadweep, and Puducherry). We also present findings for three groups of states based on their Socio-demographic Index (SDI) as calculated by GBD.^43^ This SDI is a composite indicator of development status, which ranges from 0 to 1, and is a geometric mean of the values of the indices of lag-distributed per capita income, mean education in people aged 15 years or older, and total fertility rate in people younger than 25 years. The states were categorised into three state groups based on their SDI in 2017: low SDI (≤0·53), middle SDI (0·54–0·60), and high SDI (>0·60; appendix p 27).

We report the estimated exposure levels for ambient particulate matter PM_2·5_, percentage of households using solid fuels, and ambient ozone in 2017. We estimated the deaths and DALYs attributable to air pollution, ambient particulate matter pollution and household air pollution in each state of India in 2017. We report cause-specific DALYs attributable to air pollution in India in 2017, and compared these with DALYs attributable to tobacco use for the diseases attributable to both risk factors. We estimated what the life expectancy would have been in each state of India if air pollution concentrations had been less than the theoretical minimum risk exposure level causing health loss. For this analysis, the ratio of air pollution-deleted deaths to all-cause deaths was calculated as one minus the proportion of air pollution deaths. This ratio was then used to create air pollution-deleted probability of death. Using this new probability of death, life tables were recalculated to get the life expectancies in the absence of air pollution. These computations were also done separately for ambient particulate matter pollution and household air pollution. We describe findings for ambient particulate and household air pollution in detail but not for ambient ozone pollution because this risk factor contributes only a small fraction of the health loss due to air pollution in India as well as globally. We assessed India's contribution to the global DALYs attributable to air pollution in GBD 2017.^36^

We report estimates with 95% uncertainty intervals (UIs) where relevant. UIs were based on 1000 runs of the models for each quantity of interest, with the mean regarded as the point estimate and the 2·5th and 97·5th percentiles considered the 95% UI (appendix p 15).^36^

### Role of the funding source

Some staff of the Indian Council of Medical Research are coauthors on this paper, having contributed to various aspects of the study and analysis. The other funder of the study had no role in the study design, data collection, data analysis, data interpretation, or writing of this paper. The corresponding author had full access to all the data in the study and had final responsibility for the decision to submit for publication.

## Results

**Figure 1:**
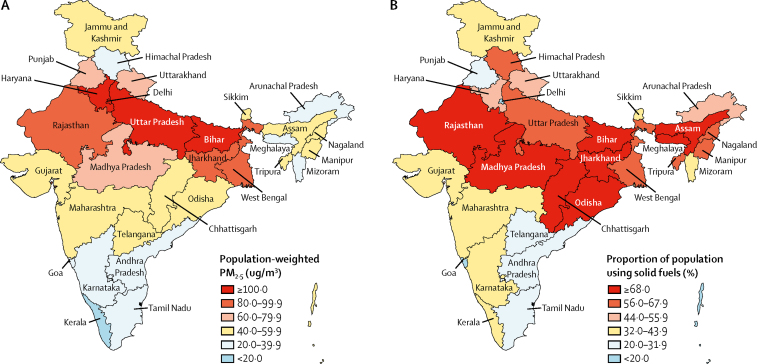
PM_2·5_ concentration and use of solid fuels in the states of India, 2017 (A) Population-weighted mean ambient air PM_2·5_ (B) Proportion of population using solid fuels.

The annual exposure to ambient particulate matter, as the population-weighted mean PM_2·5_, in India in 2017 was 89·9 μg/m^3^ (95% UI 67·0–112·0), which was one of the highest in the world (table 1; appendix p 28). The highest annual population-weighted mean PM_2·5_ in India in 2017 was in Delhi (209·0 μg/m^3^ [95% UI 120·9–339·5]), followed by Uttar Pradesh, Bihar, and Haryana in north India (range 125·7–174·7 μg/m^3^), and then in Rajasthan, Jharkhand, and West Bengal (range 81·4–93·4 μg/m^3^; figure 1; appendix p 29). Exposure was highest in the low SDI state group (125·3 μg/m^3^ [95% UI 87·5–167·3]; table 1). Of the total population in India in 2017, 42·6% of residents were exposed to mean PM_2·5_ greater than 80 μg/m^3^ and 76·8% were exposed to mean PM_2·5_ greater than 40 μg/m^3^, which is the limit recommended by the National Ambient Air Quality Standards in India.^44^ Across the states of India, the annual population-weighted mean PM_2·5_ exposure was 12·1 times greater in the state with the highest exposure than in the state with the lowest exposure in 2017.

**Table 1:** Distribution of annual mean PM_2·5_ concentration, proportion of population using solid fuels, and ozone concentration in the states of India grouped by SDI, 2017

	**Population-weighted annual mean PM**_2·5_**μg/m^3^ (95% UI)**	**Percentage of population using solid fuels (95% UI)**	**Population-weighted ozone concentration in parts per billion (95% UI)**
Low SDI states (675 million)	125·3 (87·5–167·3)	72·1 (71·1–73·0)	63·6 (63·5–63·8)
Middle SDI states (387 million)	58·7 (44·8–76·6)	46·7 (45·7–47·8)	59·0 (58·7–59·4)
High SDI states (318 million)	56·6 (44·0–71·6)	31·0 (30·0–32·1)	56·3 (55·8–56·8)
India (1380 million)	89·9 (67·0–112·0)	55·5 (54·8–56·2)	60·1 (59·9–60·2)

Population in 2017 given in parentheses. SDI=Socio-demographic Index. UI=uncertainty interval.

The proportion of the population using solid fuels in India in 2017 was 55·5% (95% UI 54·8–56·2). This proportion was highest in the low SDI state group (72·1% [71·1–73·0]; table 1); and highest in the low SDI states of Bihar, Jharkhand, and Odisha (range 76·7–81·5%), followed by Chhattisgarh, Assam, Madhya Pradesh, and Rajasthan in the low SDI state group and Meghalaya in the middle SDI state group (range 68·0–74·8%; figure 1; appendix p 29). Across the states of India, the proportion of the population using solid fuels in 2017 was 42·9 times greater in the state with the highest use than in the state with the lowest use. The annual exposure to population-weighted ambient ozone concentration in India in 2017 was 60·1 parts per billion (95% UI 59·9–60·2), with the highest exposure in the low SDI state groups (table 1).

In 2017, 1·24 million (95% UI 1·09–1·39) deaths in India were attributable to air pollution (table 2). Of the total deaths in India in 2017, 12·5% could be attributed to air pollution; this proportion was 10·8% in people younger than 70 years and 15·1% in those aged 70 years or older.^36^, ^41^ 51·4% (49·9–54·1) of the deaths attributable to air pollution in India in 2017 were in people younger than 70 years (table 2). This proportion was higher in the low SDI group than the high SDI group, but there were variations within each SDI state group. For example, in the low SDI state group, this proportion was higher in Jharkhand, Chhattisgarh, and Bihar than in the other states, and in the high SDI state group, there was a striking contrast between states, with Punjab having a much higher proportion than Kerala (table 2). Across the states of India, the proportion of deaths attributable to air pollution in 2017 was 3·1 times greater in the state with the highest proportion than in the state with the lowest proportion.

**Table 2:** Deaths attributable to air pollution, ambient particulate matter pollution, and household air pollution in the states of India, 2017

		**Death rate per 100 000 population attributable to air pollution (95% UI)**	**Number of deaths attributable to air pollution (95% UI)**	**Percentage of total deaths attributable to air pollution that were in people younger than 70 years (95% UI)**	**Number of deaths attributable to ambient particulate matter pollution (95% UI)**	**Number of deaths attributable to household air pollution (95% UI)**
**India**		**89·9 (78·7–100·4)**	**1 240 530 (1 086 200–1 385 930)**	**51·4 (49·1–54·1)**	**673 129 (551 832–793 262)**	**481 738 (393 810–580 207)**
**Low SDI states**		**95·4 (81·5–108·3)**	**643 872 (549 996–731 115)**	**53·5 (51·1–56·7)**	**340 190 (263 550–416 005)**	**258 287 (205 354–324 027)**
	Bihar	79·0 (68·5–89·3)	96 967 (84 078–109 709)	57·0 (54·0–60·3)	53 634 (34 033–71 587)	37 824 (25 054–53 047)
	Madhya Pradesh	97·0 (83·8–111·6)	83 045 (71 698–95 520)	50·0 (47·0–53·1)	37 745 (26 975–52 117)	39 895 (28 515–51 405)
	Jharkhand	69·0 (60·1–78·1)	26 486 (23 080–29 956)	59·2 (56·5–62·1)	12 053 (8629–16 445)	12 768 (9280–16 397)
	Uttar Pradesh	111·1 (87·0–131·0)	260 028 (203 701–306 568)	53·1 (50·4–56·8)	161 178 (111 757–213 041)	78 888 (50 625–113 260)
	Rajasthan	112·5 (88·6–132·8)	90 499 (71 340–106 868)	50·9 (47·9–55·3)	43 295 (28 068–59 617)	39 288 (27 444–52 551)
	Chhattisgarh	98·9 (86·5–111·9)	29 841 (26 102–33 768)	57·8 (54·9–60·7)	11 144 (7844–14 823)	17 028 (13 231–21 093)
	Odisha	65·3 (54·6–80·6)	31 118 (26 035–38 400)	54·9 (51·0–58·5)	11 985 (8004–16 865)	17 633 (13 486–22 464)
	Assam	72·3 (62·3–82·2)	25 888 (22 282–29 426)	53·1 (50·0–56·6)	9156 (6748–12 050)	14 962 (12 114–18 319)
**Middle SDI states**		**86·7 (76·3–97·7)**	**336 235 (295 958–378 769)**	**50·2 (47·8–52·9)**	**173 401 (140 417–209 827)**	**139 053 (111 735–167 916)**
	Andhra Pradesh	83·7 (65·5–105·2)	45 525 (35 629–57 235)	48·7 (45·5–52·1)	23 280 (17 188–31 262)	19 345 (13 519–25 999)
	West Bengal	93·3 (81·4–106·6)	94 534 (82 494–108 038)	50·9 (48·1–53·9)	49 882 (38 014–61 616)	38 846 (29 193–49 869)
	Tripura	91·1 (76·3–106·3)	3711 (3107–4329)	49·5 (45·9–53·7)	1627 (1236–2090)	1842 (1410–2331)
	Arunachal Pradesh	36·0 (28·9–45·4)	608 (488–766)	50·0 (46·4–54·1)	197 (124–282)	363 (270–473)
	Meghalaya	42·7 (34·3–51·7)	1440 (1157–1742)	54·8 (51·2–59·0)	520 (378–694)	847 (629–1091)
	Karnataka	94·8 (79·9–109·9)	64 333 (54 254–74 645)	49·9 (47·0–52·9)	26 311 (17 415–36 597)	33 697 (25 528–42 243)
	Telangana	65·8 (51·6–81·7)	26 000 (20 400–32 271)	50·4 (47·4–53·5)	15 239 (11 355–20 095)	8789 (5940–12 008)
	Gujarat	84·9 (70·0–99·2)	58 696 (48 429–68 625)	49·3 (46·4–52·5)	29 791 (20 117–41 188)	24 169 (17 239–31 012)
	Manipur	57·2 (46·4–69·8)	1949 (1583–2380)	50·0 (46·7–53·6)	944 (678–1269)	908 (671–1208)
	Jammu and Kashmir	75·4 (61·7–88·3)	10 476 (8579–12 265)	45·8 (43·1–48·8)	5822 (4157–7681)	3496 (2459–4680)
	Haryana	100·1 (84·5–116·6)	28 965 (24 456–33 749)	54·3 (51·9–57·1)	19 788 (14 268–25 114)	6751 (4230–10 120)
**High SDI states**		**81·9 (72·9–91·5)**	**260 421 (231 677–290 889)**	**47·5 (44·9–50·0)**	**159 538 (132 798–188 666)**	**84 398 (67 746–104 058)**
	Uttarakhand	106·4 (88·0–125·9)	12 000 (9917–14 190)	44·7 (42·1–47·8)	6959 (4524–9575)	3570 (2260–5185)
	Tamil Nadu	75·9 (63·6–90·2)	61 205 (51 249–72 725)	53·0 (50·0–56·1)	39 860 (28 617–54 082)	19 625 (13 916–25 680)
	Mizoram	52·9 (42·4–64·7)	652 (522–797)	46·0 (43·1–49·6)	339 (242–446)	243 (176–317)
	Maharashtra	86·9 (74·7–99·2)	108 038 (92 977–123 398)	44·3 (41·6–47·1)	62 677 (48 480–77 981)	36 932 (26 928–47 989)
	Punjab	86·3 (75·5–97·1)	26 594 (23 259–29 896)	58·1 (55·5–60·7)	19 178 (15 170–23 383)	6139 (4128–8543)
	Sikkim	61·5 (48·2–75·2)	413 (323–505)	43·5 (40·8–46·8)	243 (170–319)	131 (89–184)
	Nagaland	48·8 (38·8–60·5)	958 (762–1188)	50·5 (46·9–54·4)	427 (315–562)	494 (359–661)
	Himachal Pradesh	99·7 (80·2–119·1)	7485 (6022–8937)	40·9 (38·2–44·1)	3307 (2073–4602)	2986 (2080–4046)
	Union territories other than Delhi	48·5 (36·3–65·0)	1812 (1356–2425)	52·0 (48·6–55·7)	1362 (886–1973)	340 (226–485)
	Kerala	79·3 (68·2–91·3)	28 051 (24 130–32 278)	38·6 (35·3–42·0)	12 754 (10 003–16 224)	13 758 (10 834–16 961)
	Delhi	65·3 (54·4–76·9)	12 322 (10 264–14 498)	51·1 (48·7–53·5)	11 732 (9705–13 882)	52 (27–93)
	Goa	58·2 (46·9–73·7)	892 (719–1130)	42·5 (39·1–45·8)	700 (539–914)	129 (85–184)

SDI=Socio-demographic Index. UI=uncertainty interval.

The number of deaths attributable to ambient particulate matter pollution in India in 2017 was 0·67 million (95% UI 0·55–0·79) and the number attributable to household air pollution was 0·48 million (0·39–0·58; table 2). Among the low SDI states, the point estimate of the number of deaths attributable to ambient particulate matter pollution was two times higher than that of household air pollution in Uttar Pradesh and 1·4 times higher in Bihar, although with wide uncertainty ranges, consistent with the very high exposure to ambient particulate matter pollution in these states (table 2; appendix p 30). In most of the other low SDI states, however, the point estimate of the number of deaths attributable to household air pollution was higher than that of ambient particulate matter pollution, but again with wide uncertainty ranges. Delhi, in the high SDI state group, stands out as having an extreme contrast between the deaths attributable to ambient particulate matter pollution. Two other north Indian states, Haryana and Punjab, also had a higher number of deaths attributable to ambient particulate matter pollution than attributable to household air pollution. In two neighbouring high SDI states in south India, Tamil Nadu and Kerala, Tamil Nadu had twice the number of deaths attributable to ambient particulate matter pollution than to household air pollution, whereas Kerala had a similar number of deaths attributable to ambient particulate matter pollution than to household air pollution. These findings were consistent with the higher exposure levels to ambient particulate matter pollution in Tamil Nadu than in Kerala, and vice versa for household air pollution exposure.

The point estimate for the number of deaths attributable to ambient particulate matter pollution in males in India in 2017 (0·39 million [95% UI 0·32–0·46]) was 38·3% higher than for females (0·28 million [0·22–0·34]), but with some overlap in their 95% UIs (appendix p 30). By contrast, the point estimate for the number of deaths attributable to household air pollution in India in 2017 was 17·6% higher for females (0·26 million [0·21–0·31]) than for males (0·22 million [0·17–0·28]), but with considerable overlap in their 95% UIs. Although the direction of these male versus female trends was similar in most states, there were many variations between the states in the magnitude of these differences (appendix p 30).

Of the total 480·7 million (441·7–526·3) DALYs in India in 2017, 38·7 million (34·5–42·4) or 8·1% (7·1–9·0) were attributable to air pollution. 21·3 million (17·7–25·1) or 4·4% (3·7–5·3) of the total DALYs were attributable to ambient particulate matter pollution, 15·8 million (13·3–19·1) or 3·3% were attributable to household air pollution, and 2·6 million (0·9–4·2) or 0·5% (0·2–0·9) were attributable to ambient ozone pollution.^36^, ^42^ The 1·38 billion people in India in 2017 made up 18·1% of the global 7·64 billion population, but India had 38·7 million (26·2%) of the global 147·4 million DALYs attributable to air pollution in 2017.^42^

**Figure 2:**
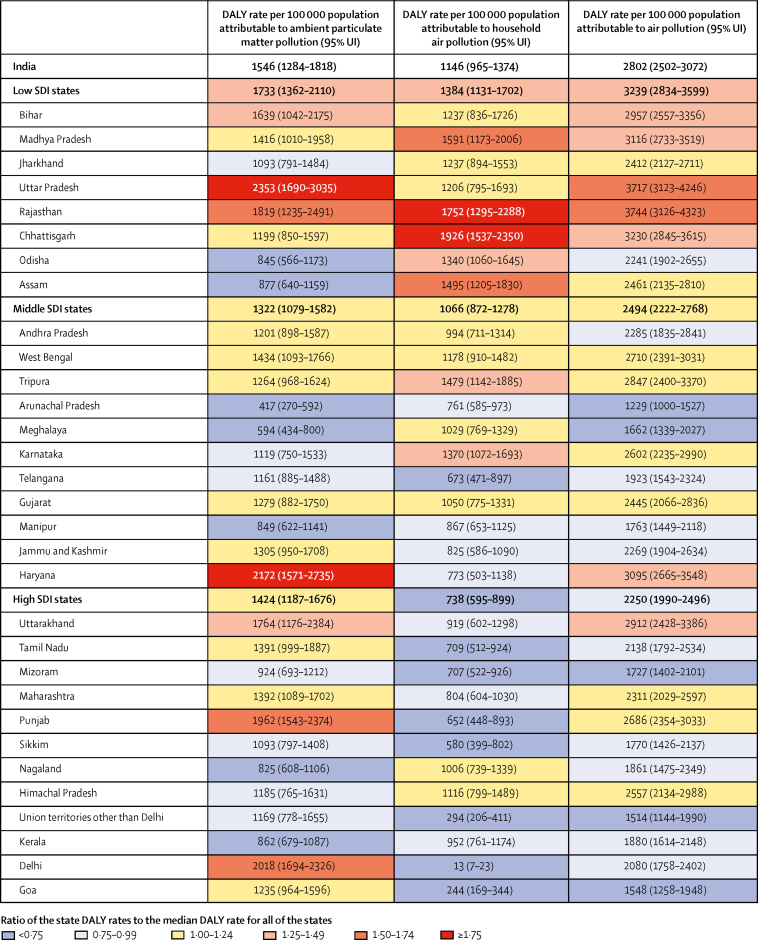
DALY rates attributable to ambient particulate matter pollution, household air pollution, and air pollution in the states of India, 2017 DALY=disability-adjusted life-year. SDI=Socio-demographic Index. UI=uncertainty interval.

The DALY rate attributable to household air pollution in 2017 was 1·9 times higher in the low SDI group than in the high SDI group and the rate attributable to ambient particulate matter was 1·4 times higher in the low SDI group than the high SDI group (figure 2). The DALY rate attributable to ambient particulate matter pollution was highest in the north Indian states of Uttar Pradesh, Haryana, Delhi, Punjab, and Rajasthan, spread across the three SDI state groups. The DALY rate attributable to household air pollution was highest in the low SDI states of Chhattisgarh, Rajasthan, Madhya Pradesh, and Assam in north and northeast India. The highest DALY rate due to household air pollution was 144·8 times the lowest rate and the highest rate due to ambient particulate matter pollution was 5·6 times the lowest. The overall DALY rate attributable to air pollution was highest in the states of Rajasthan, Uttar Pradesh, Chhattisgarh, Madhya Pradesh, Haryana, Bihar, and Uttarakhand.

**Figure 3:**
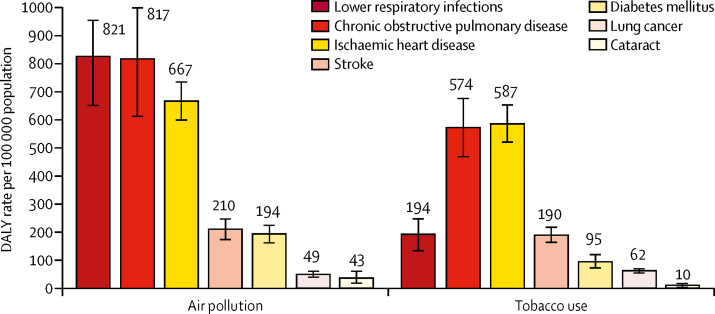
DALY rates attributable to air pollution and tobacco use in India, 2017 Error bars represent 95% uncertainty intervals. DALY=disability-adjusted life-year.

Of the total DALYs attributable to air pollution in India in 2017, the largest proportions were from lower respiratory infections (29·3%), chronic obstructive pulmonary disease (29·2%), and ischaemic heart disease (23·8%), followed by stroke (7·5%), diabetes (6·9%), lung cancer (1·8%), and cataract (1·5%). The DALY rate attributable to air pollution in India in 2017 was much higher for lower respiratory infections than the rate attributable to tobacco use (figure 3). For non-communicable diseases, including chronic obstructive pulmonary disease, ischaemic heart disease, stroke, diabetes, lung cancer, and cataract, the DALY rate attributable to air pollution was at least as high as the rate attributable to tobacco use.

If the air pollution levels in India had been lower than the theoretical minimum risk exposure levels associated with health loss, the average life expectancy in India in 2017 would have been higher by 1·7 years (95% UI 1·6–1·9; table 3), with this increase exceeding 2 years in the north Indian states of Rajasthan (2·5 years [2·0–2·8]), Uttar Pradesh (2·2 years [1·8–2·5]), and Haryana (2·1 years [1·9–2·4]). If the exposure to ambient particulate matter pollution had been lower than the minimum levels associated with health loss, the average life expectancy would have increased in India by 0·9 years (0·8–1·1), with the highest increase in Delhi (1·5 years [1·3–1·7]), Haryana (1·4 years [1·1–1·8]), Punjab (1·3 years [1·0–1·5]) and Uttar Pradesh (1·3 years [1·0–1·7]). If the exposure to household air pollution due to solid fuels had been lower than the minimum levels associated with health loss, the average life expectancy would have increased in India by 0·7 years (0·6–0·8), with the highest increase in Rajasthan (1·0 years [0·8–1·3]), Chhattisgarh (0·9 years [0·7–1·1]), and Madhya Pradesh (0·9 years [0·7–1·1]). Generally, across the states, this beneficial impact on life expectancy would have been slightly higher for males in relation to ambient particulate matter pollution and slightly higher for females in relation to household air pollution, although the UIs overlap between the two sexes (appendix p 31).

**Table 3:** Impact of air pollution on life expectancy in the states of India, 2017

	**Life expectancy at birth in 2017, years (95% UI)**	**Increase in life expectancy if air pollution concentrations were less than the minimum level causing health loss, years (95% UI)**		
		Ambient particulate matter pollution	Household air pollution	Air pollution
**India**	**69·0 (68·5–69·4)**	**0·9 (0·8–1·1)**	**0·7 (0·6–0·8)**	**1·7 (1·6–1·9)**
Bihar	69·6 (68·5–70·4)	1·0 (0·7–1·3)	0·7 (1·5–1·0)	1·9 (1·7–2·1)
Madhya Pradesh	67·1 (68·5–67·8)	0·8 (0·6–1·1)	0·9 (0·7–1·1)	1·9 (1·7–2·1)
Jharkhand	68·6 (68·5–69·2)	0·7 (0·5–0·9)	0·8 (0·6–0·9)	1·6 (1·5–1·8)
Uttar Pradesh	65·6 (68·5–66·4)	1·3 (1·0–1·7)	0·6 (0·4–0·8)	2·2 (1·8–2·5)
Rajasthan	68·2 (68·5–69·0)	1·1 (0·8–1·5)	1·0 (0·8–1·3)	2·5 (2·0–2·8)
Chhattisgarh	64·5 (68·5–65·2)	0·6 (0·4–0·7)	0·9 (0·7–1·1)	1·6 (1·4–1·8)
Odisha	68·5 (68·5–69·2)	0·4 (0·3–0·6)	0·7 (0·5–0·8)	1·2 (1·0–1·4)
Assam	66·8 (68·5–67·5)	0·5 (0·4–0·6)	0·8 (0·7–1·0)	1·5 (1·3–1·7)
Andhra Pradesh	71·0 (68·5–72·9)	0·7 (0·6–0·8)	0·6 (0·4–0·7)	1·4 (1·2–1·5)
West Bengal	70·9 (68·5–71·7)	0·9 (0·7–1·1)	0·7 (0·6–0·9)	1·7 (1·6–1·9)
Tripura	69·9 (68·5–71·2)	0·7 (0·6–0·9)	0·8 (0·7–1·0)	1·7 (1·6–1·9)
Arunachal Pradesh	70·8 (68·5–72·4)	0·3 (0·2–0·5)	0·6 (0·5–0·8)	1·1 (0·9–1·3)
Meghalaya	69·8 (68·5–71·4)	0·4 (0·3–0·5)	0·7 (0·6–0·8)	1·2 (1·1–1·4)
Karnataka	67·7 (68·5–68·4)	0·6 (0·4–0·7)	0·7 (0·6–0·9)	1·4 (1·2–1·6)
Telangana	71·5 (68·5–73·4)	0·8 (0·6–0·9)	0·4 (0·3–0·5)	1·3 (1·2–1·5)
Gujarat	70·4 (68·5–71·1)	0·8 (0·6–1·1)	0·7 (0·5–0·8)	1·7 (1·4–1·9)
Manipur	70·8 (68·5–72·2)	0·6 (0·4–0·7)	0·5 (0·4–0·7)	1·2 (1·1–1·3)
Jammu and Kashmir	72·8 (68·5–73·6)	1·1 (0·8–1·4)	0·6 (0·5–0·8)	2·0 (1·7–2·3)
Haryana	69·2 (68·5–69·9)	1·4 (1·1–1·8)	0·5 (0·3–0·7)	2·1 (1·9–2·4)
Uttarakhand	69·8 (68·5–70·5)	1·1 (0·8–1·4)	0·5 (0·4–0·7)	1·9 (1·6–2·2)
Tamil Nadu	70·5 (68·5–71·2)	0·7 (0·5–0·9)	0·3 (0·3–0·4)	1·1 (1·0–1·3)
Mizoram	70·5 (68·5–72·1)	0·6 (0·5–0·8)	0·5 (0·4–0·6)	1·3 (1·1–1·4)
Maharashtra	71·6 (68·5–72·2)	0·9 (0·7–1·0)	0·5 (0·4–0·6)	1·5 (1·3–1·7)
Punjab	72·3 (68·5–73·0)	1·3 (1·0–1·5)	0·4 (0·3–0·5)	1·8 (1·6–2·0)
Sikkim	72·5 (68·5–74·2)	0·8 (0·6–1·0)	0·4 (0·3–0·5)	1·4 (1·2–1·6)
Nagaland	70·8 (68·5–72·5)	0·5 (0·4–0·6)	0·6 (0·5–0·7)	1·2 (1·1–1·3)
Himachal Pradesh	72·3 (68·5–73·2)	0·8 (0·5–1·0)	0·7 (0·5–0·9)	1·7 (1·5–2·0)
Union territories other than Delhi	73·2 (68·5–74·9)	0·8 (0·6–1·0)	0·2 (0·1–0·3)	1·1 (1·9–1·3)
Kerala	74·6 (68·5–75·3)	0·4 (0·4–0·5)	0·5 (0·4–0·6)	1·0 (0·9–1·1)
Delhi	73·6 (68·5–74·5)	1·5 (1·3–1·7)	0·0 (0·0–0·0)	1·6 (1·4–1·8)
Goa	75·3 (68·5–76·9)	0·8 (0·6–0·9)	0·1 (0·1–0·2)	1·0 (0·9–1·1)

States are listed in increasing order of Socio-demographic Index in 2017 (appendix p 27). UI=uncertainty interval.

## Discussion

India has one of the highest annual average ambient particulate matter PM_2·5_ exposure levels in the world. In 2017, no state in India had an annual population-weighted ambient particulate matter mean PM_2·5_ less than the WHO recommended level of 10 μg/m^3^,^45^ and 77% of India's population was exposed to mean PM_2·5_ more than 40 μg/m^3^, which is the recommended limit set by the National Ambient Air Quality Standards of India. Although the use of solid fuels for cooking has been declining in India,^25^, ^26^ 56% of India's population was still exposed to household air pollution from solid fuels in 2017. Behind these high overall air pollution exposure levels in India, there is a marked variation between the states, with a 12 times difference for ambient particulate matter pollution and 43 times difference for household air pollution. The low SDI states in north India had some of the highest levels of both ambient particulate matter and household air pollution, especially Bihar, Uttar Pradesh, Rajasthan, and Jharkhand; and the middle and high SDI states Delhi, Haryana, and Punjab in north India had some of the highest ambient particulate matter pollution exposure in the country.

India had 18% of the global population in 2017, but had 26% of global DALYs attributable to air pollution. A substantial 8% of the total disease burden in India and 11% of premature deaths in people younger than 70 years could be attributed to air pollution. We estimated that 1·24 million deaths in India in 2017 could be attributed to air pollution, including 0·67 million to ambient particulate matter pollution and 0·48 million to household air pollution. Furthermore, a report has suggested that there are additional diseases attributable to air pollution that are currently not being included in the estimates of deaths attributable to air pollution in GBD, leading to underestimation of the health impact of air pollution.^46^

We estimated that life expectancy in India would have been increased by 1·7 years if the pollution levels had been lower than the minimum levels associated with health loss, including 0·9 years for ambient particulate matter pollution reduction and 0·7 years for household air pollution reduction. This potential increase in life expectancy would have been highest in some of the large less-developed states in north India that have a high dual burden of ambient particulate matter and household air pollution. Our estimate of the impact of air pollution on life expectancy in India is lower than previous reports.^34^, ^35^ One report, which applied a linear extrapolation of an estimate of life expectancy increase per unit decrease in PM_2·5_ from a US county-level study, estimated an impact of 3·4 years on life expectancy from ambient air pollution in India, including PM_2·5_ and ozone.^34^ Because the relationship between air pollution and mortality is steeper at lower levels of exposure, such as in the USA, linear extrapolations from these low PM_2·5_ concentrations to the higher concentrations in India would overestimate its impact.^46^, ^47^ Another report using a life table approach similar to the one used in our study, but which used GBD 2016 air pollution findings, estimated an adverse impact of 1·5 years on life expectancy from ambient particulate matter pollution in India.^35^ Our lower estimates of the impact of ambient particulate matter pollution using GBD 2017 findings are probably related to the improvement in GBD 2017 methods for estimating the impact of air pollution, which avoids the potential overestimation of disease burden in people exposed to both ambient particulate matter and household air pollution. This new method resulted in overall lower attribution of disease burden to air pollution in India than in GBD 2016. However, even with this reduced estimated impact, air pollution remains a leading risk factor for death and disease burden in India in 2017. It is important to note that GBD has thus far attributed diseases to air pollution for which definitive evidence of causality is available, which has led to robust estimates for the diseases that have been included, but this also results in underestimation of the overall impact of air pollution because of non-inclusion of the diseases for which the evidence is emerging but not fully established yet.^48^

It is useful to note that although air pollution is commonly thought to be associated with lung disease, a substantial 38% of the disease burden due to air pollution in India is from cardiovascular disease and diabetes. Another notable aspect of air pollution in India is its contribution to the disease burden from ischaemic heart disease, stroke, chronic obstructive pulmonary disease, and lung cancer, which are commonly associated with smoking. The DALYs for these diseases that are attributable to air pollution at the population level in India are similar to those attributable to tobacco use. Policies aimed at tobacco use control in India seem to have resulted in a decline in smoking,^27^ which is a good public health achievement that needs to be sustained. Efforts to control air pollution are also needed in India to reduce the burden of these major non-communicable diseases.

Many studies from across the world, including some from India, have provided evidence for the association of air pollution with cardiovascular and lung diseases.^1^, ^8^, ^37^, ^38^, ^46^, ^49^, ^50^ Although a large proportion of this evidence is from settings more developed than India, evidence from studies of the health impact of short-term exposure to air pollution indicate similar responses in the Indian population with those in other countries.^8^, ^17^ Evidence from a cohort study in China, which included exposure at levels similar to those in India, reported cardiovascular disease, respiratory disease, and lung cancer mortality relative risks for PM_2·5_ that are similar to those estimated from studies in high-income countries.^50^ Prospective cohort studies that have been initiated in India for studying the long-term health impact of air pollution on cardiovascular disease, respiratory disease, and birthweight are expected to provide further evidence on this topic in India.^14^, ^51^, ^52^ In brief, the available evidence indicates that the relative risks for adverse health outcomes associated with exposure to air pollution from studies worldwide can be used to estimate the health loss from air pollution in India (appendix pp 16–20).

Control of ambient particulate matter pollution requires action in several sectors and the linkage of these actions for greatest impact. Several studies have estimated the contribution of various sources to particulate matter pollution in different parts of India,^3^, ^4^, ^5^, ^6^, ^7^, ^8^, ^9^, ^10^, ^11^ which can be useful in informing the efforts that are needed to address these sources. Several government initiatives have been launched in the past few years to reduce air pollution. These include a reduction in particulate matter emissions by coal power plants and reduction in energy consumption by energy-intensive industries (Ministry of Power), setting emission standards for the brick manufacturing industry and facilitating management of agricultural residues to reduce stubble burning (Ministry of Environment), stricter vehicle emissions regulation and upgrading of vehicles to more fuel-efficient standards (Ministry of Road Transport and Highways; and Ministry of Petroleum and Natural Gas), and enhancing availability of public transport (Ministry of Urban Development).^19^, ^20^, ^53^, ^54^, ^55^ Mechanisms that help to reduce air pollution should also be included in the Smart Cities Mission launched by the Government of India.^56^ About two-thirds of the electricity in India is produced from fossil fuels, mainly coal,^57^ but India has pledged in the Paris Climate Agreement to generate 40% of its electricity from renewable sources by 2030.^58^

State-specific policies such as use of compressed natural gas by vehicles in Delhi, subsidies for alternative technologies to compost agricultural waste instead of burning it in Punjab, and mandatory use of fly ash in the construction industry within 100 km from coal or lignite thermal plants in Maharashtra could be expanded to other states to efficiently control particulate matter emissions.^8^ Another initiative is the Clean Air for Delhi Campaign launched in early 2018, which subsequently led to the launch of the National Clean Air Programme that aims to sensitise the public and enhance coordination between the implementing agencies for control of air pollution across the country.^22^, ^23^, ^24^ Other initiatives such as the Intended Nationally Determined Contributions targets to reduce particulate matter emission intensity by 33–35% by 2030, promotion of electric public transport fleets, and upgrading vehicles to Bharat Stage VI (which is equivalent to Euro-VI standard) vehicle emission standards, are also encouraging but will take some time before any substantial effect is seen.^18^, ^53^, ^59^ The very high ambient particulate matter pollution levels in north India in the winter season result in attention to this matter by the media and public with discussion often focusing on the acute health problems due to high pollution, whereas the much more important longer-term adverse health effects of chronically high pollution levels throughout the year have yet to be fully realised.^60^ More awareness needs to be created about the slow but substantial impact of ambient particulate matter and household air pollution among policy makers and the general public, which would help further enhance the air pollution control efforts in India.

Government initiatives to reduce solid fuel use for tackling household air pollution include a major scheme initiated by the Prime Minister of India in May, 2016—the Pradhan Mantri Ujjwala Yojana.^21^ This scheme had planned to provide clean and safe cooking fuel (liquefied petroleum gas) to 50 million low-income households by March, 2019, by adding 10 000 more distributors, increasing access, and covering nearly all the upfront costs of switching for low-income households. Encouragingly, the original target of 50 million households was met in August, 2018, and the government has now increased the target to reach 80 million households through this scheme with a total budget of US$1·8 billion.^61^ Liquefied petroleum gas meets the International Standards Organization and WHO recommendations, and can potentially help in achieving the WHO air quality standards within homes, but adoption and sustained use of clean fuels by households will be needed.^62^, ^63^ Income, education, and urban location have been shown to be associated with the adoption of cleaner stoves and fuels, and better understanding of the role of uninterrupted fuel availability and prices as well as household size, composition, and gender roles in decision making can help to achieve sustained use.^64^ Targeted and innovative subsidies for liquefied petroleum gas appear necessary to increase and sustain the use of clean cooking fuels, and have the potential to transform the associated expenditures into social investments.^63^, ^65^, ^66^ Furthermore, several studies report residential biomass use-related emissions to be one of the largest contributors to population-weighted ambient PM_2·5_ concentrations.^8^, ^67^, ^68^ In densely populated communities, it has also been shown that health-relevant reductions in household air pollution are best accomplished when entire communities transition to clean fuels.^69^ This provides additional justification for initiatives such as smokeless villages in the Pradhan Mantri Ujjwala Yojana.^21^

According to the WHO database of air pollution, 14 of the 15 cities with the worst air pollution in the world are in India.^70^ The experience in controlling air pollution in Mexico City and Beijing could be instructive for dealing with the extremely high pollution levels in New Delhi and other cities of India. Mexico and China have been making long-term efforts to switch to cleaner energy options, improve the application of emission-controlling technologies, promote public transport systems, promulgate policies to reduce total energy consumption, and promote environmental education and research, which attempt to address all major sources of air pollution through coordinated air quality management.^71^, ^72^, ^73^, ^74^

The general limitations associated with GBD methods for risk factors estimates were published previously.^36^ Specifically for India, the relatively low number of PM_2·5_ ground monitoring stations across the country, with none in rural areas, is a key limitation, which will be crucial to address for both air quality management and research. The expansion of automatic continuous ambient air quality monitoring stations across India in the past few years,^75^ and the proposal in the National Clean Air Programme to set up rural monitoring stations and increase the number of monitoring stations measuring PM_2·5_ across the country,^23^ are likely to strengthen the air pollution estimates in India. The scarcity of data on ozone exposure in India needs to be addressed as well. Another important area that needs strengthening is the generation of more evidence on the association of air pollution with health loss in India. Long-term cohort studies reporting adverse health effects of air pollution in India are scarce, although some are underway and expected to provide useful evidence in future; however, more are needed to strengthen this evidence. The strengths of the findings presented in this report include a comprehensive assessment of air pollution exposure in every state of India and the associated health loss using all accessible data from multiple sources, the improved GBD 2017 methods for assessing the health impact of air pollution, assessment of the impact of air pollution as part of a single GBD framework that includes all risk factors and diseases, and the substantial inputs to the analysis and interpretation of findings by a network of environmental risk factors experts in India.

In conclusion, these findings not only highlight the serious adverse health impact that is being caused by air pollution across India, but also bring into focus the large variations between the states in the exposure to air pollution and the associated health loss. The state-level findings presented in this report can serve as a useful guide to plan further interventions specific for the situation in each state. India should implement both short-term and long-term comprehensive policies and mechanisms to reduce the high levels of air pollution that pose a major threat to the long-term development of India. Encouragingly, the discussion on air pollution in India by the media, public, and other stakeholders has been increasing substantially and policy makers seem keen to address the problem.^22^, ^76^, ^77^, ^78^ This positive momentum could be boosted further by the state-specific evidence presented in this report to enhance the planning and implementation of air pollution control efforts across India in a sustainable manner. It is important to note that besides benefitting human health, the reduction of air pollution in India would also have a broader beneficial impact on other aspects of the ecosystem, including animal and plant health.
