# Supplementary material for: The impact of air pollution on deaths, disease burden, and life expectancy across the states of India: the Global Burden of Disease Study 2017
Source: Lancet Planet Health. 2019 Jan;3(1):e26–39. doi: 10.1016/S2542-5196(18)30261-4 (PMC6358127; doi:10.1016/S2542-5196(18)30261-4)
Supplement: Supplementary appendix [file mmc1.pdf]

THE LANCET
Planetary Health

Supplementary appendix

This appendix formed part of the original submission and has been peer reviewed. We post it as supplied by the authors.

Supplement to: India State-Level Disease Burden Initiative Air Pollution Collaborators. The impact of air pollution on deaths, disease burden, and life expectancy across the states of India: the Global Burden of Disease Study 2017. *Lancet Planet Health* 2018; published online Dec 6. http://dx.doi.org/10.1016/ S2542-5196(18)30261-4.

The impact of air pollution on deaths, disease burden, and life expectancy across the states of India: the Global Burden of Disease Study 2017

India State-Level Disease Burden Initiative Air Pollution Collaborators

Web Appendix

Correspondence to: Prof. Lalit Dandona, lalit.dandona@phfi.org

Table of Contents

1. GBD 2017 air pollution estimation methods 3
2. Evidence for assessing the impact of air pollution on disease burden in India 16
3. GBD 2017 India data inputs for air pollution exposure and the disease burden and deaths attributable to air pollution 21
4. Grouping of the states of India based on Socio-demographic Index, 2017 27
5. Top ten countries with the highest exposure level to ambient particulate matter pollution, 2017 28
6. Mean $PM_{2.5}$ level and proportion of population using solid fuels in each state of India, 2017 29
7. Deaths attributable to ambient particulate matter and household air pollution by sex in the states of India, 2017 30
8. Impact of air pollution on life expectancy in the states of India by sex, 2017 31

1. GBD 2017 air pollution estimation methods

The materials presented here are adapted from:
GBD 2017 Risk Factors Collaborators. Global, regional, and national comparative risk assessment of 84 behavioural, environmental and occupational, and metabolic risks or clusters of risks for 195 countries and territories, 1990–2017: a systematic analysis for the Global Burden of Disease Study 2017. *Lancet* 2018; 2018; 392: 1923–94.

A. GBD estimation process for risk factors including air pollution

The approach used in GBD 2017 for comparative risk assessment to estimate population attributable fractions for risk factors is shown in the following flowchart.

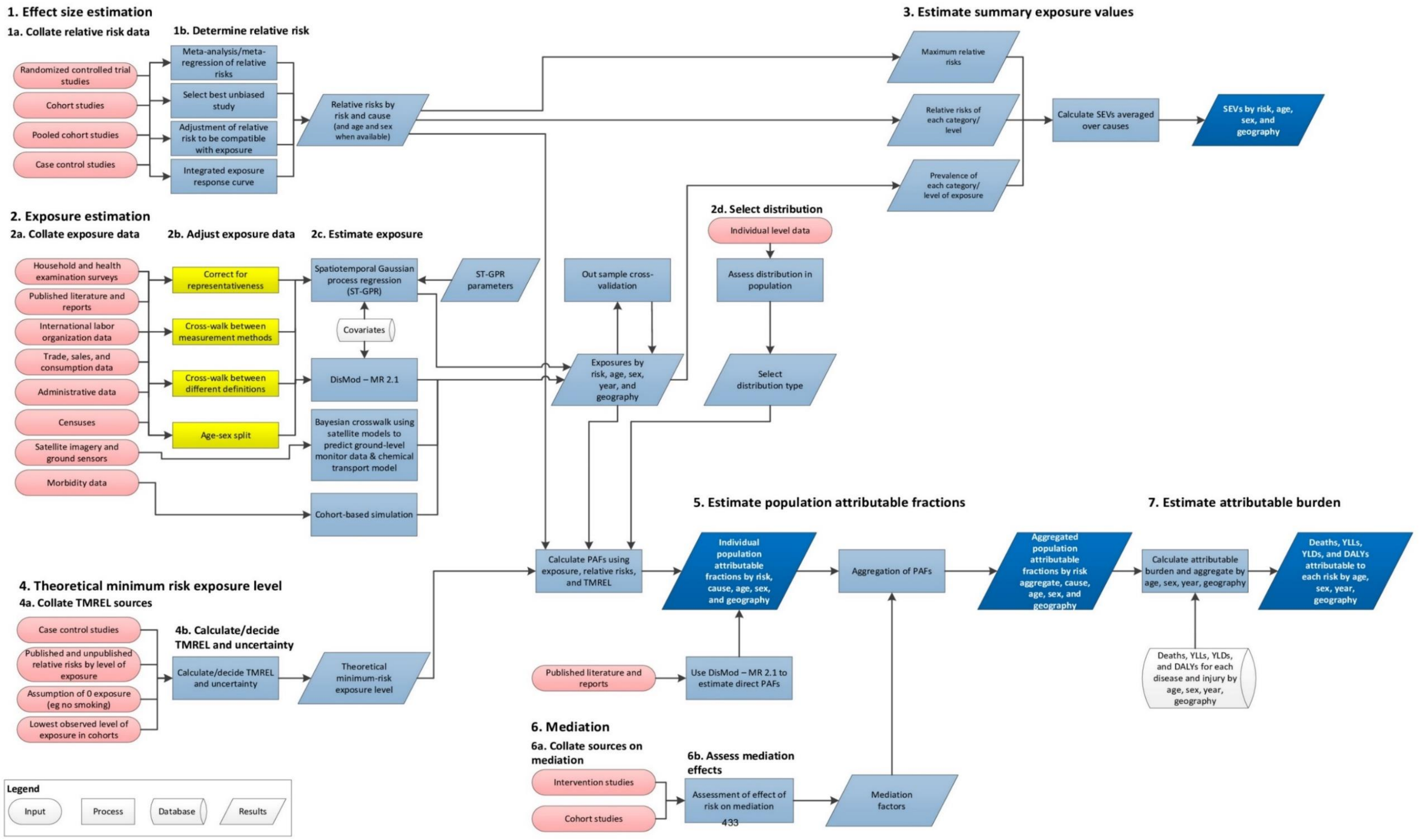

GBD is Global Burden of Disease. SEV is summary exposure value. TMREL is theoretical minimum-risk exposure level. PAF is population attributable fraction. YLL is years of life lost. YLD is years lived with disability. DALY is disability-adjusted life-year. Ovals represent data inputs, rectangular boxes represent analytical steps, cylinders represent databases, and parallelograms represent intermediate and final results.

The components of air pollution risk factor, main model types used, and the main data sources for exposure in GBD 2017 are summarised below:

Risk factor	Level	Model type	Main data source for exposure
**Air pollution**	**2**		
Particulate matter pollution	3		
Ambient particulate matter pollution	4	Regression crosswalk between grid-level fusion of satellite/chemical transport models and ground level monitoring data	Atmospheric chemical transport models, satellite measurements of aerosols in the atmosphere, data from ground-level monitoring sites
Household air pollution from solid fuels	4	ST-GPR	Population surveys and censuses
Ambient ozone pollution	3	Chemical transport model, which is an ensemble of multiple chemical transport model estimates that is bias-corrected with ground measurements	Atmospheric chemical transport models

A.1. Ambient particulate matter pollution

Exposure to ambient particulate matter pollution was defined as the population-weighted annual average mass concentration of particles with an aerodynamic diameter less than 2.5 micrometres ($PM_{2.5}$) in a cubic meter of air at a spatial resolution of 0.1° x 0.1° over the globe, which is approximately 11 x 11 km at the equator. This measurement is reported in $\mu g/m^3$. These estimates were based on multiple satellite-based aerosol optical depth data globally combined with a chemical transport model, and calibration of these with $PM_{2.5}$ data from the ground-level monitoring stations.

For the purpose of attributing disease burden to ambient particulate matter pollution, the theoretical minimum-risk exposure level (TMREL) was defined as population-weighted mean between 2.4 and 5.9 $\mu g/m^3$, bounded by the minimum and fifth percentiles of exposure distributions from outdoor air pollution (OAP) cohort studies. This uniform distribution represents the uncertainty regarding adverse effects of low-level exposure. To include the uncertainty in the TMREL, we took a random draw from the uniform distribution of the interval between 2.4 and 5.9 μg/m3 each time the population attributable burden was calculated. TMREL was defined as a uniform distribution rather than a fixed value in order to represent the uncertainty regarding the level at which the scientific evidence was consistent with adverse effects of exposure. The specific OAP cohort studies selected for this averaging were based on the criteria that their fifth percentiles were less than that of the American Cancer Society Cancer Prevention II (CPSII) cohort's fifth percentile of 8.2 based on Turner et al.[1]

The steps in the estimation of disease burden attributable to ambient particulate matter pollution are shown in the following flowchart:

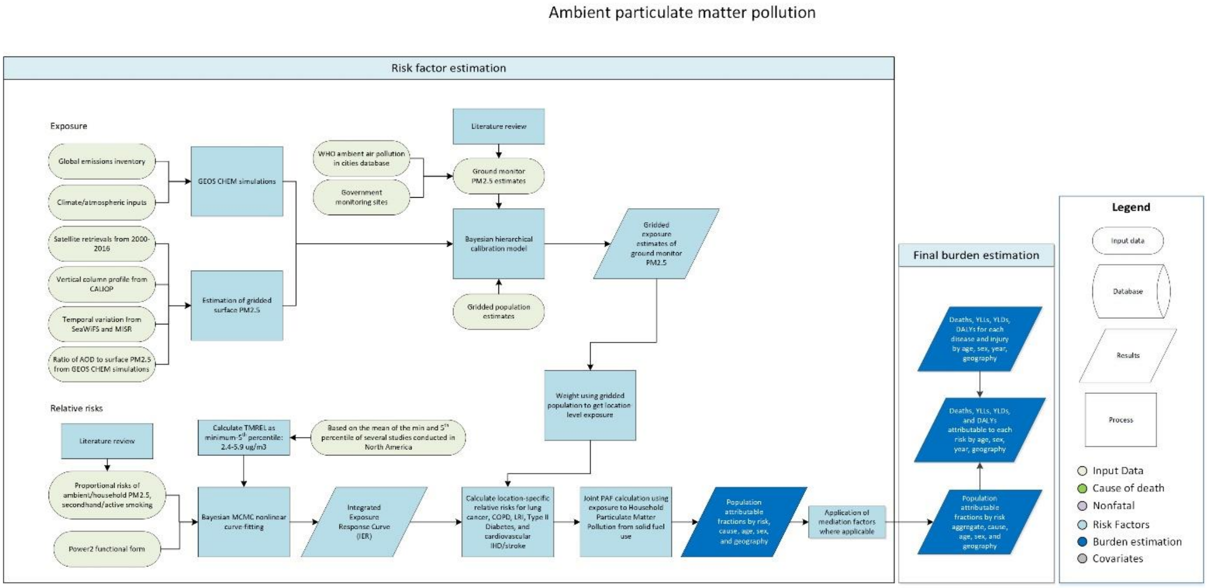

Data

The estimates of ambient $PM_{2.5}$ exposures in India were based on multiple satellite-based aerosol optical depth data combined with a chemical transport model, and calibration of these with $PM_{2.5}$ data from ground-level monitoring stations.

$PM_{2.5}$ ground measurements: More recent monitoring data from new locations were used in GBD 2017. Monitor-specific measurements (rather than city averages as reported in the WHO Air Pollution in Cities database) were used, resulting in measurements of concentrations of PM_{10} and $PM_{2.5}$ from approximately 10,000 ground monitors from 113 countries. For locations measuring only PM_{10}, $PM_{2.5}$ measurements were estimated from PM_{10}. This was performed using a locally derived conversion factor ($PM_{2.5}/PM_{10}$ ratio, for stations where measurements were available for the same year) that was estimated using population-weighted averages of location-specific conversion factors for the country or state. If country-level conversion factors were not available, the average of country-level conversion factors within a region were used. Additional information related to the ground measurements was also included where available, including monitor geo coordinates and monitor site type. Estimates in GBD 2017 included a substantially increased number of ground monitoring sites from India, which included data from 185 ground monitors for $PM_{2.5}$ and 184 monitors for PM_{10}.

Satellite-based estimates: These estimates were available at 0.1×0.1^{o} resolution (~11 x 11 km resolution at the equator) which combines aerosol optical depth retrievals from multiple satellites with the GEOS Chem chemical transport model and land use information.[2] The model to calibrate satellite-based estimates to these measurements varied smoothly over space and time in regions with many measurements.

Population data: A comprehensive set of population data on a high-resolution grid was obtained from the Gridded Population of the World (GPW v4r10) database. These data were provided on a 0.0417×0.0417^{o} resolution. Aggregation to each 0.1×0.1^{o} grid cell comprised of summing the central 3×3 population cells. As this resulted in a resolution higher than necessary, it was repeated four times, each offset by one cell in a North, South, East and West direction. The average of the resulting five quantities was used as the estimated population for each grid cell.

Chemical transport model simulations: Estimates of the sum of particulate sulphate, nitrate, ammonium and organic carbon and the compositional concentrations of mineral dust simulated using the GEOS Chem chemical transport model, and a measure combining elevation and the distance to the nearest urban land surface were available from 2000 to 2016 for each 0.1×0.1^{o} grid cell.[2]

Modelling strategy

Annual mean exposure to $PM_{2.5}$ was estimated in 5-year intervals from 1990 onward, at 0·1 × 0·1° resolution using estimates from satellites combined with a chemical transport model, surface measurements, and geographical data. We aggregated gridded exposure concentrations to national-level population-weighted means using the corresponding grid cell population value. National-level population-weighted mean concentrations and the 95% uncertainty interval (95% UI) around this mean were estimated by sampling 1000 draws of each grid cell value and its uncertainty distribution.

An updated version of the Data Integration Model for Air Quality (DIMAQ) was used for ambient particulate matter pollution modelling.[2,3] The coefficients in the calibration model were estimated for each country or state. Where data were insufficient within a country or state, information was `borrowed' from a higher aggregation (region) and if enough information was still not available from an even higher level (super region). Individual country or state level estimates were therefore based on a combination of information from the state, country, its region and super-region. This was implemented within a Bayesian Hierarchical Modelling (BHM) framework. BHMs provide an extremely useful and flexible framework in which to model complex relationships and dependencies in data. Uncertainty can also be propagated through the model allowing uncertainty arising from different components, both data sources and models, to be incorporated within estimates of uncertainty associated with the final estimates. The results of the modelling comprise a posterior distribution for each grid cell, rather than just a single point estimate, allowing a variety of summaries to be calculated. The primary outputs here were the median and 95% credible intervals for each grid cell.

The GBD 2017 model (DIMAQ-2) was updated to also include within country variation in calibrations. The model used for GBD 2017, DIMAQ-2, provides a number of substantial improvements over the initial formulation of DIMAQ. In DIMAQ, ground measurements from different years were all assumed to have been made in the primary year of interest (i.e. 2014 for GBD 2015 before extrapolation) and then regressed against values from other inputs (e.g. satellites etc.) made in that year. In the presence of changes over time therefore, and particularly in areas where no recent measurements were available, there was the possibility of mismatches between the ground measurements and other variables. In DIMAQ-2, ground measurements were matched with other inputs over time, and the possibility of the global level coefficients being allowed to vary over time, subject to smoothing that was induced by a second-order random walk process. In addition, the manner in which spatial variation can be incorporated within the model was developed: where there was sufficient data, the calibration equations can now vary (smoothly) both within and between countries, achieved by allowing the coefficients to follow (smooth) Gaussian processes. Within a geographic location where there was insufficient data, in order to produce accurate equations, information was borrowed from lower down the hierarchy and was supplemented with information from the wider region.

Due to both the complexity of the models and the size of the data, notably the number of spatial predictions that were required, recently developed techniques that perform 'approximate' Bayesian inference based on Integrated Nested Laplace Approximations (INLA) were used.[4] Computation was performed using the R interface to the INLA computational engine. Fitting the models and performing predictions for each of the 1.4 million grid cells required the use of a high performance computing cluster making use of high memory nodes.

Model development and comparison was performed using within- and out-of-sample assessment. In the evaluation, cross validation was performed using 25 combinations of training (80%) and validation (20%) datasets. Validation sets were obtained by taking a stratified random sample, using sampling probabilities based on the cross-tabulation of $PM_{2.5}$ categories (0-24.9, 25-49.9, 50-74.9, 75-99.9, 100+ $\mu g/m^3$) and super-regions, resulting in them having the same distribution of $PM_{2.5}$ concentrations and super-regions as the overall set of sites. The following metrics were calculated for each training/evaluation set combination: for model fit - R^2 and deviance information criteria (DIC, a measure of model fit for Bayesian models); for predictive accuracy - root mean squared error (RMSE) and population weighted root mean squared error.

All modelling was performed on the log-scale. The choice of which variables were included in the model was made based on their contribution to model fit and predictive ability. The following is a list of variables and model structures that were considered in developing the model:

Variable	Model structure
Continuous explanatory variables	(SAT) Estimate of $PM_{2.5}$ (in µgm-3) for 2014 from satellite remote sensing on the log scale.
	(CTM) Estimate of $PM_{2.5}$ (in µgm-3) for 2010 from the TM5 chemical transport model on the log-scale.
	(POP) Estimate of population for 2014 on the log-scale.
	(SNAOC) Estimate of the sum of sulphate, nitrate, ammonium and organic carbon simulated using the GEOS Chem chemical transport model.
	(DST) Estimate of compositional concentrations of mineral dust simulated using the GEOS Chem chemical transport model.
	(EDxDU) The log of the elevation difference between the elevation at the ground measurement location and the mean elevation within the GEOS Chem simulation grid cell multiplied by the inverse distance to the nearest urban land surface.
Discrete explanatory variables	(LOC) Binary variable indicating whether exact location of ground measurement is known.
	(TYPE) Binary variable indicating whether exact type of ground monitor is known.
	(CONV) Binary variable indicating whether ground measurement is $PM_{2.5}$ or converted from PM_{10}.
Random Effects	Grid cell random effects on the intercept to allow for multiple ground monitors in a grid cell.
	State-country-region-super-region hierarchical random effects for the intercept.
	State-country-region-super-region hierarchical random effects for the coefficient associated with SAT.
	State-country-region-super-region hierarchical random effects for the coefficient associated with the difference between estimates from CTM and SAT.
	State-country-region-super-region hierarchical random effects for the coefficient associated with POP.
	State/country level random effects for population uses a neighbourhood structure allowing specific borrowing of information from neighbouring geographies.
	Within a region, country or state level effects of SAT and the difference between SAT AND CTM were assumed to be independent and identically distributed.
	Within a super-region, region level random effects were assumed to be independent and identically distributed.
	Super-region random effects were assumed to be independent and identically distributed.
Interactions	Interactions between the binary variables and the effects of SAT and CTM.

The final model contained the following variables: SAT, POP, SNAOC, DST, EDxDU, LOC, TYPE, and CONV, together with interactions between SAT and each of LOC, TYPE and CONV. The model structure contained grid cell random effects on the intercept to allow for multiple ground monitors in a grid cell, state-country-region-super-region hierarchical random effects for intercepts and SAT and state/country level random effects for population using a neighbourhood structure allowing specific borrowing of information from neighbouring countries together with region-super-region hierarchical random effects for POP.

Satellite estimates, populations and quantities estimated using the GEOS-Chem model were available for 1990, 1995, 2000, 2005, 2010 to 2017. Population estimates for 2000, 2005, 2010, 2015 and 2020 were available from GPW version 4r10. For 1990 and 1995 data were extracted from GPW version 4r10.[3] As with populations for 2015, values for each cell for 2011 to 2017 were obtained by interpolation using natural splines with knots placed at 2000, 2005, 2010, 2015 and 2020.

These were used as inputs to DIMAQ, enabling estimates of exposures to be obtained for each of these years respectively. For 2017, estimates of exposures were obtained from predictions from locally-varying regression models.[5] For each cell a model was fit to the values within that cell over time, with a constraint placed on the rate of change between 2016 and 2017 to avoid unrealistic and/or unjustified extrapolation of trends. Measures of uncertainty were obtained by repeating the procedure for the limits of the 95% intervals, again on a cell-by-cell basis.

We estimated the burden attributable to $PM_{2.5}$ for ischaemic heart disease (IHD), stroke, lung cancer, chronic obstructive pulmonary disease (COPD), and acute lower respiratory infections (LRI). The GBD

2017 type II diabetes was added as a relative risk outcome. These were also the pollutant-outcome pairs used to estimate the ambient particulate matter pollution attributable burden. The results from all cohort studies published so far were used that reported cause-specific relative risk (RR) estimates based on measured or modelled $PM_{2.5}$ and that adjusted for potential confounding due to other major risk factors such as tobacco smoking using data for each study participant.

A recently published work assembled the evidence for the relationship between particulate matter and diabetes to generate IER curves and attributable burden estimates based on methodologies similar to those of the GBD.[6] When generating the IER for type II diabetes, all eight of the studies summarized by Bowe et al. were included in addition to the other cohorts. The resulting attributable burden estimates were remarkably similar to GBD 2017 results.

Integrated exposure-response function (IER)

IERs were developed for each cause of death to estimate the RR of mortality over the entire global range of ambient annual mean $PM_{2.5}$ concentrations using risk estimates from studies of ambient particulate matter pollution, household air pollution (HAP), and second-hand smoke exposure and active smoking. IERs assign concentrations of $PM_{2.5}$ to each type of exposure on an equivalent μg/m³ basis assuming that risk was determined by the 24-h $PM_{2.5}$ inhaled dose regardless of the exposure source. The IER was created to ascertain the shape of the dose response curve for a variety of health outcomes across a wide range of exposure to $PM_{2.5}$. The IER model was fit by integrating RR information from studies of OAP, second hand tobacco smoke, HAP, and active smoking. Because OAP studies are often performed at the lower end of the OAP range, incorporating other exposures to particulate matter enables RR estimation across the global range of exposure. These methods have been described in detail elsewhere.[7,8] Notable changes for GBD 2017 included the added OAP cohorts, the inclusion of HAP cohorts, and updated literature reviews for active smoking studies.

All published and unpublished cohorts of long-term exposure to ambient $PM_{2.5}$ and incidence or mortality due to IHD, stroke, COPD, lung cancer, and LRIs were considered. Newly published cohorts of long-term exposure to ambient $PM_{2.5}$ and incidence or mortality due to IHD, stroke, COPD, lung cancer, and LRI were added. One notable addition was the China male cohort which included mortality due to IHD, stroke, COPD, and lung cancer.[9] This study represented a higher exposure range than most of the previously incorporated studies with 5th and 95th percentile of 15.5 and 77.1 micrograms/m^3. In GBD 2017, type II diabetes which was included as a new relative risk outcome and was estimated by including all cohorts which measured long-term $PM_{2.5}$ exposure and diabetes incidence or mortality due to diabetes.

All the available cohort studies of HAP and any of the related measured outcomes were included, along with those with binary exposure data (presence or absence of solid-fuel use for cooking). To incorporate cohort studies with binary exposure data, the $PM_{2.5}$ mapping function to obtain a $PM_{2.5}$ level attributed to solid fuel use for cooking for the location-year of the study (ExpHAP) was used. The OAP exposure model was used to obtain an OAP $PM_{2.5}$ level for the location-year (ExpOAP). The study RR was used to inform the curve on the range of ExpOAP to (ExpOAP + ExpHAP).

Updated systematic reviews of literature for studies examining cigarettes smoked per day and the six IER outcomes related to particulate matter were used for the high exposure range of the curve.

Epidemiological evidence for relative risk of various disease outcomes attributable to ambient particulate matter was obtained from the following studies:

Atkinson RW, Carey IM, Kent AJ, van Staa TP, Anderson HR, Cook DG. Long-term exposure to outdoor air pollution and the incidence of chronic obstructive pulmonary disease in a national English cohort. *Occup Environ Med*.2015; 72: 42–8.
Beelen R, Hoek G, van den Brandt PA, et al. Long-term effects of traffic-related air pollution on mortality in a Dutch cohort (NLCS-AIR study). *Environ Health Perspect* 2008; 116: 196–202.
Beelen R, Stafoggia M, Raaschou-Nielsen O, et al. Long-term exposure to air pollution and cardiovascular mortality: an analysis of 22 European cohorts. *Epidemiology* 2014; 25: 368–78.
Bowe B, Xie Y, Li T, Yan Y, Xian H, Al-Aly Z. The 2016 global and national burden of diabetes mellitus attributable to $PM_{2.5}$ air pollution. *Lancet Planet Health* 2018; 2: e301–12.
Brauer M, Hoek G, Van Vliet P, et al. Air pollution from traffic and the development of respiratory infections and asthmatic and allergic symptoms in children. *Am J Respir Crit Care Med* 2002; 166: 1092–8.

Burnett RT. Cox proportional survival model hazard ratios from census year to 2011 for adults aged 25 to 89 in CanCHEC cohort. [Unpublished data]
Carey IM, Atkinson RW, Kent AJ, van Staa T, Cook DG, Anderson HR. Mortality associations with long-term exposure to outdoor air pollution in a national English cohort. *Am J Respir Crit Care Med* 2013; 187: 1226–33.
Cesaroni G, Badaloni C, Gariazzo C, et al. Long-term exposure to urban air pollution and mortality in a cohort of more than a million adults in Rome. *Environ Health Perspect* 2013; 121: 324-31.
Chen H, Burnett RT, Kwong JC, et al. Risk of incident diabetes in relation to long-term exposure to fine particulate matter in Ontario, Canada. *Environ Health Perspect* 2013; 121: 804–10.
Chen LH, Knutsen SF, Shavlik D, et al. The association between fatal coronary heart disease and ambient particulate air pollution: are females at greater risk? *Environ Health Perspect* 2005; 113: 1723-9.
Clark C, Sbihi H, Tamburic L, Brauer M, Frank LD, Davies HW. Association of long-term exposure to transportation noise and traffic-related air pollution with the incidence of diabetes: a prospective cohort study. *Environ Health Perspect* 2017; 125: 087025.
Cohen AJ, Brauer M, Burnett R, et al. Estimates and 25-year trends of the global burden of disease attributable to ambient air pollution: an analysis of data from the Global Burden of Diseases Study 2015. *Lancet* 2017; 389: 1907–18.
Coogan PF, White LF, Yu J, et al. $PM_{2.5}$ and Diabetes and Hypertension Incidence in the Black Women's Health Study. *Epidemiology* 2016; 27: 202–10.
Gan WQ, FitzGerald JM, Carlsten C, Sadatsafavi M, Brauer M. Associations of ambient air pollution with chronic obstructive pulmonary disease hospitalization and mortality. *Am J Respir Crit Care Med* 2013; 187: 721–7.
Hansen AB, Ravnskjær L, Loft S, et al. Long-term exposure to fine particulate matter and incidence of diabetes in the Danish nurse cohort. *Environ Int* 2016; 91: 243–50.
He D, Wu S, Zhao H, et al. Association between particulate matter 2.5 and diabetes mellitus: a meta-analysis of cohort studies. *J Diabetes Investig* 2017; 8: 687–96.
Hertz-Picciotto I, Baker RJ, Yap P-S, et al. Early childhood lower respiratory illness and air pollution. *Environ Health Perspect* 2007; 115: 1510–8.
Hoek G, Krishnan RM, Beelen R, et al. Long-term air pollution exposure and cardio- respiratory mortality: a review. *Environ Health* 2013; 12: 43.
Honda T, Pun VC, Manjourides J, Suh H. Associations between long-term exposure to air pollution, glycosylated hemoglobin and diabetes. *Int J Hyg Environ Health* 2017; 220: 1124–32.
Huang F, Pan B, Wu J, Chen E, Chen L. Relationship between exposure to $PM_{2.5}$ and lung cancer incidence and mortality: a meta-analysis. *Oncotarget* 2017; 8: 43322–31.
Hystad P, Demers PA, Johnson KC, Carpiano RM, Brauer M. Long-term residential exposure to air pollution and lung cancer risk. *Epidemiology* 2013; 24: 762–72.
Karr C, Lumley T, Schreuder A, et al. Effects of subchronic and chronic exposure to ambient air pollutants on infant bronchiolitis. *Am J Epidemiol* 2007; 165: 553–60.
Karr CJ, Rudra CB, Miller KA, et al. Infant exposure to fine particulate matter and traffic and risk of hospitalization for RSV bronchiolitis in a region with lower ambient air pollution. *Environ Res* 2009; 109: 321–7.
Katanoda K, Sobue T, Satoh H, et al. An association between long-term exposure to ambient air pollution and mortality from lung cancer and respiratory diseases in Japan. *J Epidemiol* 2011; 21: 132–43.
Lepeule J, Laden F, Dockery D, Schwartz J. Chronic exposure to fine particles and mortality: an extended follow-up of the Harvard six cities study from 1974 to 2009. *Environ Health Perspect* 2012; 120: 965–70.
Lipsett MJ, Ostro BD, Reynolds P, et al. Long-term exposure to air pollution and cardiorespiratory disease in the California teachers study cohort. *Am J Respir Crit Care Med* 2011; 184: 828–35.
MacIntyre EA., Gehring U, Molter A, et al. Air pollution and respiratory infections during early childhood: an analysis of 10 European birth cohorts within the ESCAPE project. *Environ Health Perspect* 2014; 122: 107–13.
Mehta S, Shin H, Burnett R, North T, Cohen AJ. Ambient particulate air pollution and acute lower respiratory infections: a systematic review and implications for estimating the global burden of disease. *Air Qual Atmos Health* 2013; 6: 69–83.
Miller KA, Siscovick DS, Sheppard L, et al. Long-term exposure to air pollution and incidence of cardiovascular events in women. *N Engl J Med* 2007; 356: 447–58.
Næss Ø, Nafstad P, Aamodt G, Claussen B, Rosland P. Relation between concentration of air pollution and cause-specific mortality: four-year exposures to nitrogen dioxide and particulate matter pollutants in 470 neighbourhoods in Oslo, Norway. *Am J Epidemiol* 2007; 165: 435–43.
Newby DE, Mannucci PM, Tell GS, et al. Expert position paper on air pollution and cardiovascular disease. *Eur Heart J* 2015; 36: 83–93b.

Park SK, Adar SD, O'Neill MS, et al. Long-term exposure to air pollution and type 2 diabetes mellitus in a multiethnic cohort. *Am J Epidemiol* 2015; 181: 327–36.
Parker JD, Kravets N, Vaidyanathan A. Particulate matter air pollution exposure and heart disease mortality risks by race and ethnicity in the United States. *Circulation* 2018; 137: 1688–97.
Pinault L, Tjepkema M, Crouse DL, et al. Risk estimates of mortality attributed to low concentrations of ambient fine particulate matter in the Canadian community health survey cohort. *Environ Health* 2016; 15: 18.
Puett RC, Hart JE, Schwartz J, Hu FB, Liese AD, Laden F. Are particulate matter exposures associated with risk of type 2 diabetes? *Environ Health Per*spect 2011; 119: 384–9.
Puett RC, Hart JE, Yanosky JD, et al. Chronic fine and coarse particulate exposure, mortality, and coronary heart disease in the Nurses' Health Study. *Environ Health Perspect* 2009; 117: 1697–701.
Raaschou-Nielsen O, Andersen ZJ, Beelen R, et al. Air pollution and lung cancer incidence in 17 European cohorts: prospective analyses from the European Study of Cohorts for Air Pollution Effects (ESCAPE). *Lancet Oncol* 2013; 14: 813–22.
Raaschou-Nielsen O, Beelen R, Wang M, et al. Particulate matter air pollution components and risk for lung cancer. *Environ Int* 2016; 87: 66–73.
Scheers H, Jacobs L, Casas L, Nemery B, Nawrot TS. Long-term exposure to particulate matter air pollution is a risk factor for stroke: meta-analytical evidence. *Stroke* 2015; 46: 3058–66.
Thurston GD, Ahn J, Cromar KR, et al. Ambient particulate matter air pollution exposure and mortality in the NIH-AARP diet and health cohort. *Environ Health Perspect* 2016; 124: 484–90.
To T, Zhu J, Villeneuve PJ, et al. Chronic disease prevalence in women and air pollution: a 30-year longitudinal cohort study. *Environ Int* 2015; 80: 26–32.
Tseng E, Ho W-C, Lin M-H, Cheng T-J, Chen P-C, Lin H-H. Chronic exposure to particulate matter and risk of cardiovascular mortality: cohort study from Taiwan. *BMC Public Health* 2015; 15: 936.
Turner MC, Jerrett M, Pope CA, et al. Long-term ozone exposure and mortality in a large prospective study. *Am J Respir Crit Care Med* 2016; 193: 1134–42.
Villeneuve PJ, Weichenthal SA, Crouse D, et al. Long-term exposure to fine particulate matter air pollution and mortality among Canadian women. *Epidemiology* 2015; 26: 536–45.
Weichenthal S, Hoppin JA, Reeves F. Obesity and cardiovascular health effects of fine particulate air pollution. *Obesity (Silver Spring)* 2014; 22: 1580–9.
Weinmayr G, Hennig F, Fuks K, et al. Long-term exposure to fine particulate matter and incidence of type 2 diabetes mellitus in a cohort study: effects of total and traffic-specific air pollution. *Environ Health* 2015; 14: 53.
Wong CM, Lai HK, Tsang H, et al. Satellite-based estimates of long-term exposure to fine particles and association with mortality in elderly Hong Kong residents. *Environ Health Perspect* 2015; 123: 1167–72.
Yin P, Brauer M, Cohen A, et al. Long-term fine particulate matter exposure and nonaccidental and cause-specific mortality in a large national cohort of Chinese men. *Environ Health Perspect* 2017; 125: 117002.

The IER has the mathematical form:

$$IER(\beta,z)=1+\alpha\times(1-e^{-\beta(z-z_{cf})_+^{\gamma}})$$

where z is the level of $PM_{2.5}$ and z_{cf} is the TMREL, below which no additional risk is assumed, with

$$(z-z_{cf})_+=(z-z_{cf})$$

if z is greater than z_{cf} and zero otherwise. Here, 1 + α is the maximum risk, β is the ratio of the IER at low to high concentrations, and γ is the power of $PM_{2.5}$ concentration. Epidemiological evidence suggests that the RRs for IHD and stroke decline with age. We modified the particulate matter source-specific RR for both IHD and stroke mortality and applied this age modification to the RRs, fitting the IER model for each age group separately. Observed RRs were related to the IER within a Bayesian framework using the STAN fitting algorithm. Given the true values of the four parameters (α, β, γ, z_{cf}), we assumed that the logarithm of each study's observed RR was normally distributed, with mean defined by the IER and variance given by the square of the observed SE of the study-specific log-relative risk estimate plus an additional variance term for each of the four sources on $PM_{2.5}$ exposure (OAP, second-hand smoke, HAP, and active smoking).

It is important to recognize the inherent limitations of the IER approach. The use of various sources to construct a risk curve assumes an equitoxicity of particles, consistent with evaluations by US EPA and WHO. However, current evidence suggests there are differences in health impact by source, size, and

chemical composition. This is seen when comparing studies of ambient and household particulate matter. As this body of evidence grows, this strategy will be continually re-examined for the integrated exposure-response curve. For now, the IER is a practical solution to fill gaps in the literature where we do not have sufficient evidence such as household air pollution exposures and ambient in highly polluted areas.

Additionally, currently the exposure concentrations used for both second-hand smoking and active smoking data points when fitting the IER were contrasted with the TMREL and do not take into account ambient particulate matter pollution. In future iterations of fitting the curve, the alternate approaches will be tested, including a similar approach to HAP, allowing each data point to inform the curve on the range of ExpOAP to (ExpOAP + ExpAS/SHS).

Relative risk and proportional population-attributable fraction (PAF) approach

For GBD 2017, a new approach was developed to use the IER for obtaining PAFs for both OAP and HAP. Previously relative risks for both exposures were obtained from the IER as a function of exposure and relative to the same TMREL. Were you to reduce one of these risk factors, however, the other would remain. In GBD 2017, RRs were estimated from the output of the IER curve. Everyone is exposed to some level of OAP, but only a proportion of the population in each location-year use solid cooking fuel and were exposed to HAP. For the proportion of the population not exposed to HAP the RR was obtained using RROAP = IER(z = ExpOAP), and the PAF was calculated at each grid-cell. These PAFs were population weighted and aggregated up to each location.

For the proportion of the population exposed to both OAP and HAP, we calculated a joint RR from the IER by RROAP+HAP = IER(z = ExpOAP+ExpHAP). This joint RR was used to calculate a joint PAF for each 0.1 x 0.1 degree grid cell. For each grid cell the joint PAF was proportioned based on the proportion of exposure due to OAP and HAP, respectively. Thus the PAF of both the exposure were mutually exclusive of each other in GBD 2017. The table below indicates the equations used to calculate proportional PAFs:

PAF	Population not exposed to HAP	Population exposed to HAP
OAP	PAF_{OAP}	$(Exp_{OAP}/(Exp_{OAP}+Exp_{HAP}))*PAF_{OAP+HAP}$
HAP	0	$(Exp_{HAP}/(Exp_{OAP}+Exp_{HAP}))*PAF_{OAP+HAP}$

A 1000 predicted values of the IER for each $PM_{2.5}$ concentration were calculated based on the posterior distributions of (α, β, γ) and the prespecified uniform distribution of TMREL to characterise uncertainty in the estimates of the IER. The mean of the 1000 IER predictions at each concentration was used as the central estimate, with uncertainty defined by 95% UIs.

A.2. Household air pollution

Exposure to HAP from solid fuels in GBD analysis was defined as the proportion of households using solid cooking fuels. The definition of solid fuel in this analysis includes coal, wood, charcoal, dung, and agricultural residues. The household exposure to solid fuels was converted to average $PM_{2.5}$ exposures from solid fuel use for different household members based on studies measuring 24-hour kitchen and living area $PM_{2.5}$ concentrations in households, and estimating this for men, women and children.

For cataract where the RR were extracted based on direct epidemiological evidence, TMREL was defined such that no households would report using solid fuel as their primary cooking fuel. For the other health outcomes that utilized evidence based on IER, TMREL was defined as a uniform distribution between 2.4 and 5.9 ug/m^3. To include the uncertainty in the TMREL, we took a random draw from the uniform distribution of the interval between 2.4 and 5.9 μg/m^3 each time the population attributable burden was calculated.

The steps in the estimation of disease burden attributable to household air pollution are shown in the following flowchart:

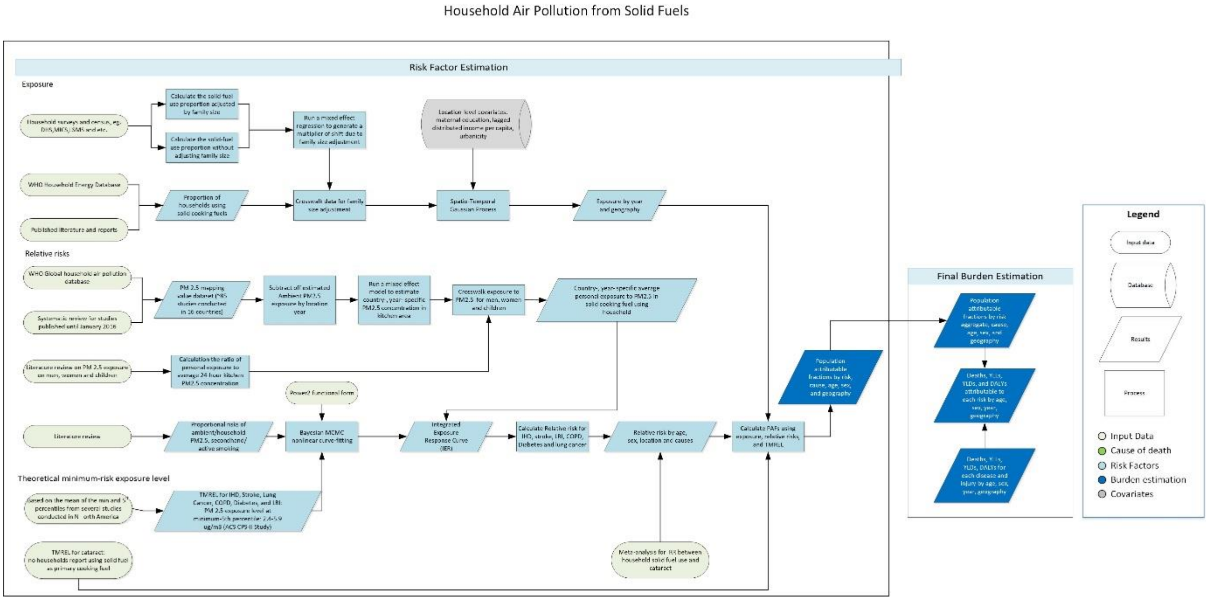

Data

There are many data sources on HAP from solid fuel use in India include national health surveys such as the National Family Health Survey and the District Level Household Survey, nationwide surveys of the National Sample Survey Organisation, and the Census of India, as well as other published and unpublished epidemiological studies.

Globally, data were extracted from the standard multi-country survey series such as Demographic and Health Surveys, Living Standards Measurement Surveys, Multiple Indicator Cluster Surveys, and World Health Surveys, as well as country-specific survey series. To fill the gaps of data in surveys and censuses, we also downloaded and updated HAP estimates from WHO Energy Database and extracted from literature through systematic review. Each nationally or sub-nationally representative data point provided an estimate for the percentage of households using solid cooking fuels. Estimates for the usage of solid fuels for non-cooking purpose were excluded, i.e. heating and primary fuels for lighting. The HAP database, with estimates from 1980 to 2017 contained about 680 studies form 150 countries.

In GBD 2017, the model was updated to estimate the individual exposure to $PM_{2.5}$ over and above ambient levels due to the use of solid cooking fuel. This was done by subtracting off the estimated ambient level $PM_{2.5}$ for the location-year of each study in the database before inputting them into the model. By doing this we derive at independent estimates for $PM_{2.5}$ exposure due to ambient and household solid fuel use. The average $PM_{2.5}$ exposures from solid fuel use for different household members were derived from studies measuring 24-hour kitchen and living area $PM_{2.5}$ concentrations in households, and estimating this for men, women and children separately.

These exposures were cross-walked to men, women, and children by generating the ratio of each group's mean exposure to the overall mean personal exposure. The resulting location, year, sex, and age specific $PM_{2.5}$ exposure values were used as inputs in the IER and attributable burden calculation process.

Modelling strategy

HAP was modelled at household level using a three-step modelling strategy that uses linear regression, spatiotemporal regression and Gaussian Process Regression (ST-GPR). The first step was a mixed-effect linear regression of logit-transformed proportion of households using solid cooking fuels. The linear model contains maternal education, proportion of population living in urban areas, and lagged-distributed income as covariates and has nested random effect by GBD region, and GBD super region respectively. Description of the full ST-GPR process is available in the GBD 2017 risk factors capstone paper (Lancet 2018; 392: 1923–94).

A variety of combinations of socioeconomic and environmental covariates in different transformation format were tested by running mixed-effect models with exposure data. The final list of covariates

included in the exposure model were maternal education, proportion of population living in urban area, and lagged-distributed income since they proved to be the strongest predictors.

The disease-outcomes paired with HAP include LRI, stroke, IHD, COPD, lung cancer, and cataract. For GBD 2017, type II diabetes was included as a new outcome of HAP. The RRs of all outcomes, with the exception of cataracts, were generated by using IER, for which a new approach was adopted in GBD 2017, as described above in the section on ambient particulate matter pollution modelling. The RR for cataracts were extracted from a meta-analysis and was 2.47 with 95% (1.61, 3.73).[10] GBD currently only estimates cataracts as an outcome for females.

In order to use the IER curve, the exposure to particulate matter with diameter of less than 2.5 micrometres ($PM_{2.5}$) must be estimated. A mapping model relying on a database of almost 90 studies which measures $PM_{2.5}$ exposure in households using solid cooking fuel was utilised. Using socio-demographic index and study-level factors as covariates, the exposure was predicted for all location-years.

Epidemiological evidence for relative risk of various disease outcomes attributable to household air pollution was obtained from the following studies:

Alam DS, Chowdhury MAH, Siddiquee AT, et al. Adult cardiopulmonary mortality and indoor air pollution: A 10-year retrospective cohort study in a low-income rural setting. *Glob Heart* 2012; 7: 215–21.
Burnett RT, Pope CA, Ezzati M, et al. An integrated risk function for estimating the global burden of disease attributable to ambient fine particulate matter exposure. *Environ Health Perspect* 2014; 122: 397–403.
Dherani M, Pope D, Mascarenhas M, Smith KR, Weber M, Bruce N. Indoor air pollution from unprocessed solid fuel use and pneumonia risk in children aged under five years: a systematic review and meta-analysis. *Bull World Health Organ* 2008; 86: 390–398C.
Fatmi Z, Coggon D. Coronary heart disease and household air pollution from use of solid fuel: a systematic review. *Br Med Bull* 2016; ll8: 91–109.
Jary H, Simpson H, Havens D, et al. Household air pollution and acute lower respiratory infections in adults: a systematic review. *Plos One* 2016; 11: e0167656.
Kim C, Seow WJ, Shu X-O, et al. Cooking coal use and all-cause and cause-specific mortality in a prospective cohort study of women in Shanghai, China. *Environ Health Perspect* 2016; 124: 1384–9.
Kurmi OP, Arya PH, Lam K-BH, Sorahan T, Ayres JG. Lung cancer risk and solid fuel smoke exposure: a systematic review and meta-analysis. *Eur Respir J* 2012; 40: 1228–37.
Mitter SS, Vedanthan R, Islami F, et al. Household fuel use and cardiovascular disease mortality: Golestan cohort study. *Circulation* 2016; 133: 2360–9.
Smith KR, Bruce N, Balakrishnan K, et al. Millions dead: how do we know and what does it mean? Methods used in the comparative risk assessment of household air pollution. *Annu Rev Public Health* 2014; 35: 185–206.
Smith KR, McCracken JP, Weber MW, et al. Effect of reduction in household air pollution on childhood pneumonia in Guatemala (RESPIRE): a randomised controlled trial. *Lancet* 2011; 378: 1717–26.
West S, Bates M, Lee J, et al. Is household air pollution a risk factor for eye disease? *Int J Environ Res Public Health* 2013; 10, 5378–98.
Yu K, Qiu G, Chan K-H, et al. Association of solid fuel use with risk of cardiovascular and all-cause mortality in rural China. *JAMA* 2018; 319: 1351–61.

A.3. Ambient ozone pollution

Exposure to ozone pollution was defined as the seasonal (6-month period with highest mean) 8 hour daily maximum ozone concentrations, measured in parts per billion (ppb). This was an update from the previous exposure metric in accordance with an update of the American Cancer Society Cancer Prevention Study II (ACS CPS-II).[1] Ozone exposure estimates in GBD 2017 incorporated a new comprehensive global ozone measurement database (Tropospheric Ozone Assessment Report).[11]

For the purpose of attributing disease burden to ambient ozone pollution, the theoretical minimum-risk exposure level was defined as population-weighted mean between 29.1 and 35.7 ppb, bounded by the minimum and fifth percentiles of exposure distributions from ambient ozone pollution cohort studies. To include the uncertainty in the TMREL, we took a random draw from the uniform distribution of the interval between 29.1 and 35.7 ppb each time the population attributable burden was calculated.

The steps in the estimation of disease burden attributable to ambient ozone pollution are shown in the following flowchart:

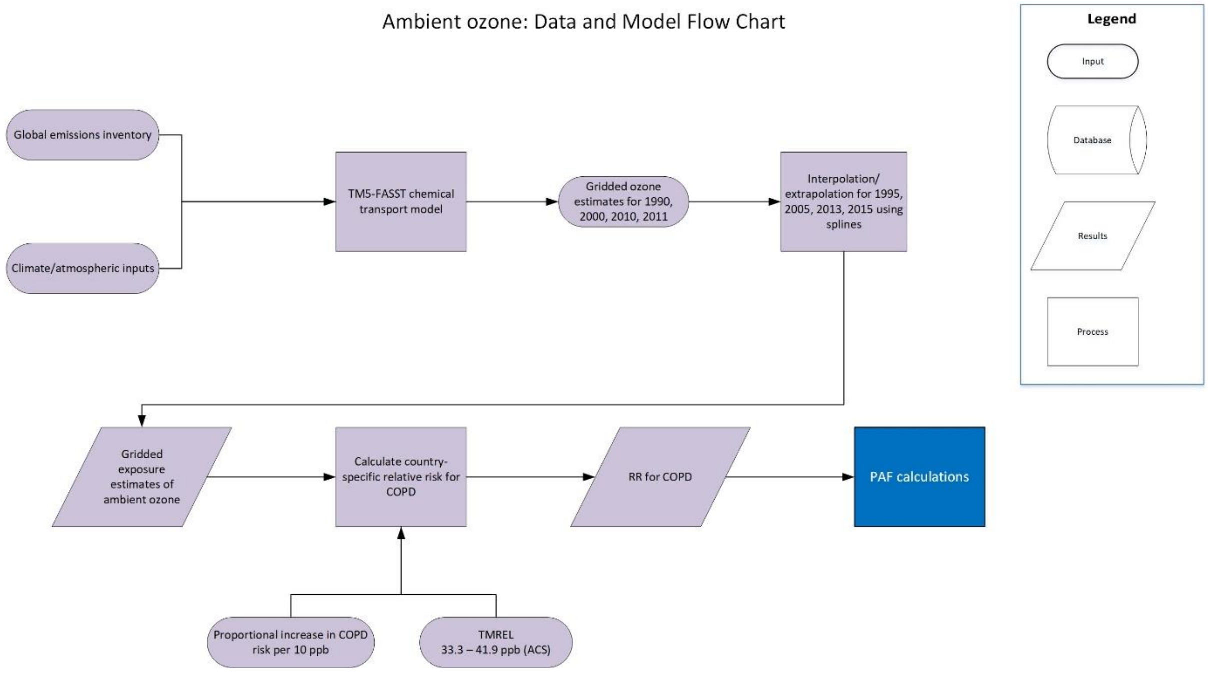

Data

Previously, exposure estimates were based on a chemical transport model with no measurement database or evaluation. In GBD 2017, exposure estimates incorporated a new comprehensive ozone measurement database (Tropospheric Ozone Assessment Report).[11] This enabled a continent-specific weighted blend of 6 chemical transport models with grid cell level bias correction. The use of ground measurements also enabled the incorporation of error estimation. The output of this model was a global raster of ozone exposure which was a summary for the years 2008-2014.[12] This global database included measurements from four sites in India.

Modelling strategy

To estimate ozone concentrations over time, the trend from the former GBD model for 1990, 2000, and 2010 and cubic splines for 1995, 2005, and 2011, were used after applying an adjustment for the difference in trends between the previous (1 hour daily maximum) and current (8 hours daily maximum) metrics. Annualised rate of change was used to predict for the years 2012-2017.

For GBD 2017, cohort studies from Canada, the UK, and the US that measured COPD mortality with ambient ozone pollution were included. The RR was estimated to be 1.06, 95% C.I. (1.02, 1.10) per 10 ppb of ozone exposure.

Epidemiological evidence for relative risk of various disease outcomes attributable to ambient ozone pollution was obtained from the following studies:

Burnett RT. Cox proportional survival model hazard ratios from census year to 2011 for adults aged 25 to 89 in CanCHEC cohort. [Unpublished data]
Carey IM, Atkinson RW, Kent AJ, van Staa T, Cook DG, Anderson HR. Mortality associations with long-term exposure to outdoor air pollution in a national English cohort. *Am J Respir Crit Care Med* 2013; 187: 1226–33.
Turner MC, Jerrett M, Pope CA, et al. Long-term ozone exposure and mortality in a large prospective study. *Am J Respir Crit Care Med* 2016; 193: 1134–42.
Weichenthal S, Pinault LL, Burnett RT. Impact of oxidant gases on the relationship between outdoor fine particulate air pollution and nonaccidental, cardiovascular, and respiratory mortality. *Sci Rep* 2017; 7: 16401.

A.4. References

1. Turner MC, Jerrett M, Pope CA III, et al. Long-term ozone exposure and mortality in a large prospective study. *Am J Respir Crit Care Med* 2016; 193: 1134–42.

2. Shaddick G, Thomas M, Amini H, et al. Data integration for the assessment of population exposure to ambient air pollution for global burden of disease assessment. *Environ Sci Technol* 2018; 52: 9069–78.

3. Shaddick G, Thomas ML, Jobling A, et al. Data integration model for air quality: a hierarchical approach to the global estimation of exposures to ambient air pollution. *J R Stat Soc Ser C Appl Stat* 2018; 67: 231–53.

4. Rue H, Martino S, Chopin N. Approximate Bayesian inference for latent Gaussian models by using integrated nested Laplace approximations. *J R Stat Soc Ser B Stat Methedol* 2009; 71:319–92.

5. Cleveland WS, Devlin SJ. Locally weighted regression: an approach to regression analysis by local fitting. *J Am Stat Assoc* 1988; 83: 596–610.

6. Bowe B, Xie Y, Li T, Yan Y, Xian H, Al-Aly Z. The 2016 global and national burden of diabetes mellitus attributable to PM2.5 air pollution. *Lancet Planet Health* 2018; 2: e301–12.

7. Cohen AJ, Brauer M, Burnett R, et al. Estimates and 25-year trends of the global burden of disease attributable to ambient air pollution: an analysis of data from the Global Burden of Diseases Study 2015. *Lancet* 2017; 389: 1907–18.

8. Burnett RT, Pope CA III, Ezzati M, et al. An integrated risk function for estimating the global burden of disease attributable to ambient fine particulate matter exposure. *Environ Health Perspect* 2014; 122: 397–403.

9. Yin P, Brauer M, Cohen A, et al. Long-term fine particulate matter exposure and nonaccidental and cause-specific mortality in a large national cohort of Chinese men. *Environ Health Perspect* 2017; 125: 117002.

10. Smith KR, Bruce N, Balakrishnan K, *et al.* Millions dead: how do we know and what does it mean? Methods used in the comparative risk assessment of household air pollution. *Annu Rev Public Health* 2014; 35: 185–206.

11. Schultz MG, Schröder S, Lyapina O, et al. Tropospheric ozone assessment report: database and metrics data of global surface ozone observations. *Elem Sci Anth* 2017; 5: 58.

12. Chang K-L, Cooper OR, West JJ, et al. A new method (M3Fusion-v1) for combining observations and multiple model output for an improved estimate of the global surface ozone distribution. *Geosci Model Dev Discuss* 2018; in review https://doi.org/10.5194/gmd-2018-183

B. Uncertainty intervals

Point estimates for each quantity of interest were derived from the mean of the draws, while 95% uncertainty intervals (UIs) were derived from the 2.5th and 97.5th percentiles of the 1000 draw level values. Uncertainty in the estimation is attributable to sample size variability within data sources, different availability of data by age, sex, year, or location, and cause specific model specifications. We determined UIs for components of cause-specific estimation based on 1000 draws from the posterior distribution of cause-specific mortality by age, sex, and location for each year included in the GBD 2017 analysis. Similarly, for non-fatal estimates if there was a change in disease estimates between locations or over time that was in the same direction in more than 950 of the 1000 samples we report it as significant. With this approach, uncertainty could be quantified and propagated into the final quantities of interest.

2. Evidence for assessing the impact of air pollution on disease burden in India

Extensive literature provides evidence to show that the adverse effects of exposure to air pollution seen in other parts of the world are also occurring in India. Two reports have previously summarized the evidence on the impact of air pollution on disease burden in India.[1,2] The following is a summary of this impact, partially adapted from these two reports, and updated with recent evidence.

There are many studies from India on the health effects of air pollution including ambient particulate matter and household air pollution. Some studies have reported increases in health-related outcomes such as acute respiratory illness and emergency room visits for cardiorespiratory conditions related to short-term exposure to air pollution.[3,4] A number of studies have reported acute health effects associated with episodic extreme air pollution events such as crop burning, use of fireworks during Diwali, and in critically polluted areas within large cities.[4-9] Some studies have found association between ambient particulate matter pollution and reduced lung function with increasing risk of developing chronic respiratory symptoms in both children and adults.[10,11] These risk estimates for health outcomes associated with short-term exposure to air pollution from Indian studies are generally similar to the estimates from studies in other parts of the world.

Time-series studies in India have also examined the association of short-term exposure to ambient particulate matter PM_{10} with all-cause mortality in the cities of Chennai, Delhi, and Ludhiana.[12-14] Studies using similar methods have also been reported from other Indian cities.[15-21] The estimated changes in the rates of all-cause mortality associated with short-term particulate matter exposure in these studies are similar to the estimates reported by several studies in cities in several countries of Asia, Europe and North America.[22-24]

Furthermore, some Indian studies have corroborated the broader global evidence for pathophysiological effects of air pollution that may underlie the development of chronic non-communicable respiratory and cardiovascular disease. These Indian studies report findings that air pollution has been associated with a range of underlying effects, including cytopathological changes, airway inflammation and oxidative stress.[25-31]

Importantly, some cohort studies have been recently initiated in India to assess the long-term adverse health impact of exposure to air pollution.[32-34] These include studies in different parts of India to assess long-term health effects of air pollution on cardiovascular disease, respiratory disease, and birth weight. Initial findings from the rural-urban cohort study in Tamil Nadu supported by the Indian Council of Medical Research indicates that the risk estimates for birth weight and lung function from exposure to air pollution obtained from continuous exposure–response modelling are remarkably consistent with the meta-analysis estimates from studies on ambient particulate matter and household air pollution elsewhere.[32,35-37]

Due to the absence of findings from long-term air pollution exposure epidemiology studies in India so far, estimates of the association of ambient $PM_{2.5}$ with mortality from chronic respiratory and cardiovascular disease obtained from long-term exposure cohort studies conducted in North America and Western Europe have been used to estimate disease burden associated with air pollution in India.[1,38] It is important to note that recent evidence from a cohort study in China, which included exposure at levels comparable to those observed in India, has reported that the relative risks for cardiovascular disease, chronic respiratory disease and lung cancer mortality from exposure to $PM_{2.5}$ are comparable to those estimated from studies in high-income countries.[39]

Since 1980, numerous epidemiological studies have examined health effects associated with household air pollution exposures in India, especially among women and children.[40] Most epidemiological studies in India assessing the association of household air pollution with health outcomes used qualitative indicators to characterize exposure, such as type of fuel used, involvement in cooking, or proximity to a stove. Several studies have shown strong evidence of the association of use of solid cooking fuels with acute lower respiratory infections in children under five, chronic obstructive lung disease in women and other health outcomes including lung cancer, cataracts, asthma and tuberculosis.[41] Several Indian studies are currently included in systematic reviews and meta-analyses used by the GBD efforts to estimate household air pollution-related risks for chronic obstructive pulmonary disease, lung cancer, lower respiratory tract infection, and cataract.[42-55] Studies in India have also found increased airway inflammation and oxidative stress when women are exposed to biomass smoke.[56,57] A number of household air pollution studies in India have also reported associations of residential biomass fuel use with increases in a range of additional health outcomes, including low birth weight, preterm births, stillbirths, asthma, other cancers and tuberculosis.[58-68] Studies have shown the association of different health outcomes including acute lower respiratory infections with long-term individual exposure measures of household air pollution on children using continuous exposure-response functions.[38,69-71]

Based on the above evidence, it seems quite reasonable to use the relative risks for adverse health outcomes from long-term exposure to $PM_{2.5}$ available from worldwide studies to estimate the impact of air pollution on disease burden in India as has been done in GBD 2017.

References

1. Ministry of Health and Family Welfare, Government of India. Report of the steering committee on air pollution and health related issues. New Delhi, 2015 https://mohfw.gov.in/sites/default/files/5412023661450432724_0.pdf
2. GBD MAPS Working Group. Burden of disease attributable to major air pollution sources in India. Special Report 21. Boston: Health Effects Institute, 2018 https://www.healtheffects.org/publication/gbd-air-pollution-india
3. Bladen WA. Relationship between acute respiratory illness and air pollution in an Indian industrial city. *J Air Pollut Control Assoc* 1983; 33: 226–7.
4. Pande JN, Bhatta N, Biswas D, et al. Outdoor air pollution and emergency room visits at a hospital in Delhi. *Indian J Chest Diseases Allied Sci* 2002; 44: 13–9.
5. Awasthi A, Singh N, Mittal S, Gupta PK, Agarwal R. Effects of agriculture crop residue burning on children and young on PFTs in North West India. *Sci Total Environ* 2010; 408: 4440–5.
6. Gupta S, Agarwal R, Mittal SK. Respiratory health concerns in children at some strategic locations from high PM levels during crop residue burning episodes. *Atmos Environ* 2016; 137: 127–134.
7. Pal R, Mahima GA, Singh C, Tripathi A, Singh RB. The effects of fireworks on ambient air and possible impact on cardiac health, during Deepawali festival in north India. *World Heart J* 2014, 5: 21–32.
8. Kumar R, Nagar JK, Kumar H, et al. Association of indoor and outdoor air pollutant level with respiratory problems among children in an industrial area of Delhi, India. *Arch Environ Occup Health* 2007; 62: 75–80.
9. Siddique S, Banerjee M, Ray MR, Lahiri T. Air pollution and its impact on lung function of children in Delhi, the capital city of India. *Water Air Soil Pollut* 2010; 212: 89–100.
10. Siddique S, Ray MR, Lahiri T. Effects of air pollution on the respiratory health of children: a study in the capital city of India. *Air Qual Atmos Health* 2011; 4: 95–102.
11. Kumar R, Goel N, Gupta N, Singh K, Nagar S, Mittal J. Indoor air pollution and respiratory illness in children from rural India: a pilot study. *Indian J Chest Dis Allied Sci* 2014; 56: 79–83.
12. Balakrishnan K, Ganguli B, Ghosh S, et al. Part 1. Short-term effects of air pollution on mortality: results from a time-series analysis in Chennai, India. *Res Rep Health Eff Inst* 2011; 157: 7–44.
13. Kumar R, Sharma SK, Thakur JS, Lakshmi PV, Sharma MK, Singh T. Association of air pollution and mortality in the Ludhiana city of India: a time-series study. *Indian J Public Health* 2010; 54: 98–103.
14. Rajarathnam U, Seghal M, Nairy S, et al. Part 2. Time-series study on air pollution and mortality in Delhi. *Res Rep Health Eff Inst* 2011; 157: 47–74.
15. Cropper ML, Simon NB, Alberini A, Arora S, Sharma PK. The health benefits of air pollution control in Delhi. *Amer J Agri Econ* 1997; 79: 1625–1629.
16. Shah J, Nagpal T. Urban air quality management strategy in Asia: Greater Mumbai report (technical paper no: 381). Washington: World Bank, 1977 http://documents.worldbank.org/curated/en/587831468772744677/Urban-air-quality-management-strategy-in-Asia-Greater-Mumbai-report.
17. Kandlikar M, Ramachandran G. The causes and consequences of particulate air pollution in urban India: a synthesis of the science. *Annu Rev Energ Environ* 2000; 25: 629–684.
18. Neema P, Goyal SK. Estimation of health impacts due to PM_{10} in major Indian cities. In: Air pollution: Health and Environmental Impacts. Boca Raton: CRC Press, 2010.
19. Guttikunda SK, Jawahar P. Urban air pollution and co-benefits analysis for Indian cities - Pune, Chennai, Indore, Ahmedabad, Surat, and Rajkot. New Delhi: Urban Emissions.Info (Ed.), SIM-air Working Paper Series, 39–2012.

20. Dholakia HH, Bhadra D, Garg A. Short term association between ambient air pollution and mortality and modification by temperature in five Indian cities. *Atmos Environ* 2014; 99: 168–174.

21. Maji S, Ahmed S, Siddiqui WA, Ghosh S. Short term effects of criteria air pollutants on daily mortality in Delhi, India. *Atmos Environ* 2017; 150: 210–19.

22. Samet JM, Zeger SL, Dominici F, et al. The national morbidity, mortality, and air pollution study. Part II: morbidity and mortality from air pollution in the United States. *Res Rep Health Eff Inst* 2000; 94:5-70; discussion 71-9.

23. Anderson HR, Atkinson RW, Peacock JL, Marston L, Konstantinou K. Meta-analysis of time-series studies and panel studies of particulate matter (PM) and ozone (O3): report of a WHO Task Group. Bonn: World Health Organization; 2004.

24. Wong C-M, Vichit-Vadakan N, Kan H, Qian Z. Public health and air pollution in Asia (PAPA): a multicity study of short-term effects of air pollution on mortality. *Environ Health Perspect* 2008; 116: 1195–1202.

25. Basu C, Ray M, Lahiri T. Traffic-related air pollution in Calcutta associated with increased respiratory symptoms and impaired alveolar macrophage activity. *J Environ Poll* 2001; 8: 187–195.

26. Roy S, Roy MR, Basu C, Lahiri P, Lahiri T. Abundance of siderophages in sputum: indicator of an adverse lung reaction to air pollution. *Acta Cytol* 2001; 45: 958–64.

27. Ray MR, Mukherjee S, Roychoudhury S, et al. Platelet activation, upregulation of cd11b/cd18 expression on leukocytes and increase in circulating leukocyte-platelet aggregates in Indian women chronically exposed to biomass smoke. *Human Exp Tox* 2006; 25: 627–35.

28. Central Pollution Control Board, Ministry of Environment, Forests and Climate Change, Government of India. Epidemiological study on effect of air pollution on human health (adults) in Delhi (Environmental Health Series: EHS/1.2008). http://cpcb.nic.in/cpcbold/upload/NewItems/NewItem_161_Adult.pdf

29. Gurjar BR, Molina LT, Ojha C, editors. Air pollution: health and environmental impacts, 1st edn. Boca Raton: CRC Press, 2010.

30. Roychoudhury S, Mondal NK, Mukherjee S, Dutta A, Siddique S, Ray MR. Activation of protein kinase B (PKB/Akt) and risk of lung cancer among rural women in India who cook with biomass fuel. *Toxicol Appl Pharmacol* 2012; 259: 45–53.

31. Dutta A, Ray MR, Banerjee A. Systemic inflammatory changes and increased oxidative stress in rural Indian women cooking with biomass fuels. *Toxicol Appl Pharmacol* 2012; 261: 255–62.

32. Balakrishnan K, Sambandam S, Ramaswamy P, et al. Establishing integrated rural-urban cohorts to assess air pollution-related health effects in pregnant women, children and adults in Southern India: an overview of objectives, design and methods in the Tamil Nadu Air Pollution and Health Effects (TAPHE) study. *BMJ Open* 2015; 5: e008090.

33. Tonne C, Salmon M, Sanchez M, et al. Integrated assessment of exposure to PM2.5 in South India and its relation with cardiovascular risk: design of the CHAI observational cohort study. *Int J Hyg Environ Health* 2017; 220: 1081–8.

34. Arku RE, Birch A, Shupler M, Yusuf S, Hystad P, Brauer M. Characterizing exposure to household air pollution within the Prospective Urban Rural Epidemiology (PURE) study. *Environ Int* 2018; 114: 307–17.

35. Balakrishnan K, Ghosh S, Thangavel G, et al. Exposures to fine particulate matter ($PM_{2.5}$) and birthweight in a rural-urban, mother-child cohort in Tamil Nadu, India. *Environ Res* 2018; 161: 524–31.

36. Amegah AK, Quansah R, Jaakkola JJK. Household air pollution from solid fuel use and risk of adverse pregnancy outcomes: a systematic review and meta-analysis of the empirical evidence. *PLoS One* 2014; 9: e113920.

37. Sun X, Luo X, Zhao C, et al. The associations between birth weight and exposure to fine particulate matter (PM2.5) and its chemical constituents during pregnancy: a meta-analysis. *Environ Pollut* 2016; 211: 38–47.

38. Burnett RT, Pope CA, Ezzati M, et al. An integrated risk function for estimating the global burden of disease attributable to ambient fine particulate matter exposure. *Environ Health Perspect* 2014; 122: 397–403.

39. Yin P, Brauer M, Cohen A, et al. Long-term fine particulate matter exposure and nonaccidental and cause-specific mortality in a large national cohort of Chinese men. *Environ Health Perspect* 2017; 125: 117002.

40. Balakrishnan K, Ramaswamy P, Sambandam S, et al. Air pollution from household solid fuel combustion in India: an overview of exposure and health related information to inform health research priorities. *Glob Health Action* 2011; 4: 5638.

41. Smith KR, Sumi M, Maeusezahl-Feuz M. Indoor air pollution from household use of solid fuels. In: Comparative quantification of health risks: global and regional burden of disease attributable to selected major risk factors. Geneva: World Health Organization, 2004: 1435–93.

42. Pandey MR, Neupane RP, Gautam A, Shrestha IB. Domestic smoke pollution and acute respiratory infections in a rural community of the hill region of Nepal. *Environ Int* 1989; 15: 337–40.

43. Mohan M, Sperduto RD, Angra SK, et al. India–US case-control study of age-related cataracts. India–US Case-Control Study Group. *Arch Opthalmol* 1989; 107: 670–76.

44. Qureshi K. Domestic smoke pollution and prevalence of chronic bronchitis/asthma in a rural area of Kashmir. *Indian J Chest Diseases Allied Sci* 1994; 36: 61–72.

45. Dutt D, Srinivasa D, Rotti S, Sahai A, Konar D. Effect of indoor air pollution on the respiratory system of women using different fuels for cooking in an urban slum of Pondicherry. *Nat Medical J India* 1996; 9: 113–7.

46. Badrinath S, Sharma T, Biswas J, Srinivas V. A case control study of senile cataract in a hospital based population. *Indian J Ophthalmol* 1996; 44: 213–17.

47. Behera D. An analysis of effect of common domestic fuels on respiratory function. *Indian J Chest Dis Allied Sci* 1997; 39: 235–43.

48. Mishra V, Retherford RD. Cooking smoke increases the risk of acute respiratory infection in children. *Nat Fam Health Surv Bull* 1997; 1–4.

49. Sreenivas V, Prabhakar A, Badrinath S, et al. A rural population based case-control study of senile cataract in India. *J Epidemiol* 1999; 9: 327–36.

50. Zodpey SP, Ughade SN. Exposure to cheaper cooking fuels and risk of age-related cataract in women. *Indian J Occup Environ Med* 1999; 3: 159–61.

51. Gupta D, Boffetta P, Gaborieau V, Jindal S. Risk factors of lung cancer in Chandigarh, India. *Ind J Med Res* 2001; 113: 142–50.

52. Mishra V, Smith KR, Retherford RD. Effects of cooking smoke and environmental tobacco smoke on acute respiratory infections in young Indian children. *Pop Environ* 2005; 26: 375–96.

53. Saha A, Kulkarni P, Shah A, Patel M, Saiyed H. Ocular morbidity and fuel use: an experience from India. *Occup Environ Med* 2005; 62: 66–9.

54. Jindal S, Aggarwal A, Chaudhry K, et al. A multicentric study on epidemiology of chronic obstructive pulmonary disease and its relationship with tobacco smoking and environmental tobacco smoke exposure. *Indian J Chest Dis Allied Sci* 2006; 48: 23–9.

55. Sapkota A, Gajalakshmi V, Jetly D, Roychowdhury S, Dikshit R. Indoor air pollution from solid fuels and risk of hypopharyngeal/laryngeal and lung cancers: a multicentric case-control study from India. *Int J Epidemiol* 2008; 37: 321–28.

56. Banerjee A, Mondal N, Das D, Ray M. Neutrophilic inflammatory response and oxidative stress in premenopausal women chronically exposed to indoor air pollution from biomass burning. *Inflammation* 2012; 35: 671–83.

57. Dutta A, Roychoudhury S, Chowdhury S, Ray M. Changes in sputum cytology, airway inflammation and oxidative stress due to chronic inhalation of biomass smoke during cooking in premenopausal rural Indian women. *Int J Hyg Environ Health* 2013; 216: 301–8.

58. Po JYT, FitzGerald JM, Carlsten C. Respiratory disease associated with solid biomass fuel exposure in rural women and children: Systematic review and meta-analysis. *Thorax* 2011; 66: 232–39.

59. Mavalankar D, Trivedi C, Gray R. Levels and risk factors for perinatal mortality in Ahmedabad, India. *Bull World Health Organ* 1991; 69: 435–42.

60. Mishra VK, Retherford RD, Smith KR. Biomass cooking fuels and prevalence of tuberculosis in India. *Int J Infecti Dis* 1999; 3: 119–29.

61. Mishra V. Effect of indoor air pollution from biomass combustion on prevalence of asthma in the elderly. *Environ Health Perspect* 2003; 111: 71–8.

62. Dherani M, Pope D, Mascarenhas M, Smith KR, Weber M, Bruce N. Indoor air pollution from unprocessed solid fuel use and pneumonia risk in children aged under five years: a systematic review and meta-analysis. *Bull World Health Organ* 2008; 86: 390–4.

63. Tielsch J, Katz J, Thulasiraj R, et al. Exposure to indoor biomass fuel and tobacco smoke and risk of adverse reproductive outcomes, mortality, respiratory morbidity and growth among newborn infants in south India. *Int J Epidemiol* 2009; 38: 1351–63.

64. Kolappan C, Subramani R. Association between biomass fuel and pulmonary tuberculosis: a nested case-control study. *Thorax* 2009; 64: 705–8.

65. Kurmi OP, Semple S, Simkhada P, Smith WC, Ayres JG. COPD and chronic bronchitis risk of indoor air pollution from solid fuel: a systematic review and meta-analysis. *Thorax* 2010; 65: 221–8.

66. Stern-Nezer J. Chronic obstructive pulmonary disease and biomass fuel combustion: systematic review and meta-analysis. California: Stanford University 2010.

67. Pope DP, Mishra V, Thompson L, et al. Risk of low birth weight and stillbirth associated with indoor air pollution from solid fuel use in developing countries. *Epidemiol Rev* 2010; 32: 70–81.

68. Lakshmi PV, Virdi NK, Thakur JS, Smith KR, Bates MN, Kumar R. Biomass fuel and risk of tuberculosis: a case-control study from northern India. *J Epidemiol Comm Health* 2012; 66: 457–61.

69. Smith K, McCracken J, Weber Mea. Effect of reduction in household air pollution on childhood pneumonia in guatemala (respire): a randomised controlled trial. *Lancet* 2011; 378: 1717–26.

70. Balakrishnan K, Ghosh S, Ganguli B, et al. State and national household concentrations of PM2.5 from solid cookfuel use: results from measurements and modeling in India for estimation of the global burden of disease. *Environ health* 2013; 12: 77.

71. Smith KR, Bruce N, Balakrishnan K, et al. Millions dead: how do we know and what does it mean? Methods used in the comparative risk assessment of household air pollution. *Annu Rev Public Health* 2014; 35: 185–206.

3. GBD 2017 India data inputs for air pollution exposure and the disease burden and deaths attributable to air pollution

Alam DS, Chowdhury MAH, Siddiquee AT, et al. Adult cardiopulmonary mortality and indoor air pollution: A 10-year retrospective cohort study in a low-income rural setting. Glob Heart 2012; 7: 215–21.
Atkinson RW, Carey IM, Kent AJ, van Staa TP, Anderson HR, Cook DG. Long-term exposure to outdoor air pollution and the incidence of chronic obstructive pulmonary disease in a national English cohort. Occup Environ Med 2015; 72: 42–8.
Atkinson RW, Cohen A, Mehta S, Anderson HR. Systematic review and meta-analysis of epidemiological time-series studies on outdoor air pollution and health in Asia. Air Qual Atmos Health 2012; 5: 383–391.
Badrinath S, Sharma T, Biswas J, Srinivas V. A case control study of senile cataract in a hospital based population. Indian J Ophthalmol 1996; 44: 213–217.
Balakrishnan K, Sambandam S, Ramaswamy P, Mehta S, Smith KR. Exposure assessment for respirable particulates associated with household fuel use in rural districts of Andhra Pradesh, India. J Expo Anal Environ Epidemiol 2004; 14(Suppl 1): S14-25.
Balakrishnan K, Sankar S, Parikh J, et al. Daily average exposures to respirable particulate matter from combustion of biomass fuels in rural households of southern India. Environ Health Perspect 2002; 110: 1069-75.
Beelen R, Hoek G, van den Brandt PA, et al. Long-term effects of traffic-related air pollution on mortality in a Dutch cohort (NLCS-AIR study). Environ Health Perspect 2008; 116: 196–202.
Beelen R, Stafoggia M, Raaschou-Nielsen O, et al. Long-term exposure to air pollution and cardiovascular mortality: an analysis of 22 European cohorts. Epidemiology 2014; 25: 368–78.
Behera D. An analysis of effect of common domestic fuels on respiratory function. Indian J Chest Dis Allied Sci 1997; 39:235–243.
Bowe B, Xie Y, Li T, Yan Y, Xian H, Al-Aly Z. The 2016 global and national burden of diabetes mellitus attributable to PM2.5 air pollution. Lancet Planet Health 2018; 2: e301–12.
Brauer M, Hoek G, Van Vliet P, et al. Air pollution from traffic and the development of respiratory infections and asthmatic and allergic symptoms in children. Am J Respir Crit Care Med 2002; 166: 1092–8.
Burnett RT, Pope CA, Ezzati M, et al. An integrated risk function for estimating the global burden of disease attributable to ambient fine particulate matter exposure. Environ Health Perspect 2014; 122: 397–403.
Burnett RT. Cox proportional survival model hazard ratios from census year to 2011 for adults aged 25 to 89 in CanCHEC cohort. [Unpublished data]
Carey IM, Atkinson RW, Kent AJ, van Staa T, Cook DG, Anderson HR. Mortality associations with long-term exposure to outdoor air pollution in a national English cohort. Am J Respir Crit Care Med 2013; 187: 1226–33.
Central Pollution Control Board (CPCB), Ministry of Environment, Forest and Climate Change, Government of India. India - National Air Quality Monitoring Programme Data 2010. New Delhi, India: CPCB.
Central Pollution Control Board (CPCB), Ministry of Environment, Forest and Climate Change, Government of India. India - National Air Quality Monitoring Programme Data 2011. New Delhi, India: CPCB.
Central Pollution Control Board (CPCB), Ministry of Environment, Forest and Climate Change, Government of India. India - National Air Quality Monitoring Programme Data 2012. New Delhi, India: CPCB.
Central Pollution Control Board (CPCB), Ministry of Environment, Forest and Climate Change, Government of India. India - National Air Quality Monitoring Programme Data 2013. New Delhi, India: CPCB.
Central Pollution Control Board (CPCB), Ministry of Environment, Forest and Climate Change, Government of India. India - National Air Quality Monitoring Programme Data 2014. New Delhi, India: CPCB.
Central Pollution Control Board (CPCB), Ministry of Environment, Forest and Climate Change, Government of India. India - National Air Quality Monitoring Programme Data 2015. New Delhi, India: CPCB.
Central Pollution Control Board (CPCB), Ministry of Environment, Forest and Climate Change, Government of India. India - National Air Quality Monitoring Programme Data 2016. New Delhi, India: CPCB.
Cesaroni G, Badaloni C, Gariazzo C, et al. Long-term exposure to urban air pollution and mortality in a cohort of more than a million adults in Rome. Environ Health Perspect 2013; 121: 324-31.
Chen H, Burnett RT, Kwong JC, et al. Risk of incident diabetes in relation to long-term exposure to fine particulate matter in Ontario, Canada. Environ Health Perspect 2013; 121: 804–10.
Chen LH, Knutsen SF, Shavlik D, et al. The association between fatal coronary heart disease and ambient particulate air pollution: are females at greater risk? Environ Health Perspect 2005; 113: 1723-9.
Chen Y, Ebenstein A, Greenstone M, Hongbin L. Evidence on the impact of sustained exposure to air pollution on life expectancy from China's Huai River policy. Proc Natl Acad Sci 2013; 110: 12936–12941
Clark C, Sbihi H, Tamburic L, Brauer M, Frank LD, Davies HW. Association of long-term exposure to transportation noise and traffic-related air pollution with the incidence of diabetes: a prospective cohort study. Environ Health Perspect 2017; 125: 087025.
Cohen AJ, Brauer M, Burnett R, et al. Estimates and 25-year trends of the global burden of disease attributable to ambient air pollution: an analysis of data from the Global Burden of Diseases Study 2015. Lancet 2017; 389: 1907–18.

Coogan PF, White LF, Yu J, et al. PM2.5 and Diabetes and Hypertension Incidence in the Black Women's Health Study. Epidemiology 2016; 27: 202–10.

Dherani M, Pope D, Mascarenhas M, Smith KR, Weber M, Bruce N. Indoor air pollution from unprocessed solid fuel use and pneumonia risk in children aged under five years: a systematic review and meta-analysis. Bull World Health Organ 2008; 86: 390–398C.

Dutt D, Srinivasa D, Rotti S, Sahai A, Konar D. Effect of indoor air pollution on the respiratory system of women using different fuels for cooking in an urban slum of Pondicherry. Nat Medical J India 1996; 9:113–117.

Embassy of the United States - New Delhi, United States Environmental Protection Agency. United States Mission India NowCast Air Quality Data 2014. New Delhi, India: Embassy of the United States.

Fatmi Z, Coggon D. Coronary heart disease and household air pollution from use of solid fuel: a systematic review. Br Med Bull 2016; ll8: 91–109.

Gan WQ, FitzGerald JM, Carlsten C, Sadatsafavi M, Brauer M. Associations of ambient air pollution with chronic obstructive pulmonary disease hospitalization and mortality. Am J Respir Crit Care Med 2013; 187: 721–7.

Global Burden of Disease Health Financing Collaborator Network, Institute for Health Metrics and Evaluation (IHME). Global Development Assistance for Health, Government, Prepaid Private, and Out-of-Pocket Health Spending 1995-2014. Seattle, United States: IHME; 2017.

Gupta D, Boffetta P, Gaborieau V, Jindal S. Risk factors of lung cancer in Chandigarh, India. Ind J Med Res. 2001; 113:142–150.

Hansen AB, Ravnskjær L, Loft S, et al. Long-term exposure to fine particulate matter and incidence of diabetes in the Danish nurse cohort. Environ Int 2016; 91: 243–50.

He D, Wu S, Zhao H, et al. Association between particulate matter 2.5 and diabetes mellitus: a meta-analysis of cohort studies. J Diabetes Investig 2017; 8: 687–96.

Hertz-Picciotto I, Baker RJ, Yap P-S, et al. Early childhood lower respiratory illness and air pollution. Environ Health Perspect 2007; 115: 1510–8.

Hoek G, Krishnan RM, Beelen R, et al. Long-term air pollution exposure and cardio- respiratory mortality: a review. Environ Health 2013; 12: 43.

Honda T, Pun VC, Manjourides J, Suh H. Associations between long-term exposure to air pollution, glycosylated hemoglobin and diabetes. Int J Hyg Environ Health 2017; 220: 1124–32.

Huang F, Pan B, Wu J, Chen E, Chen L. Relationship between exposure to PM2.5 and lung cancer incidence and mortality: a meta-analysis. Oncotarget 2017; 8: 43322–31.

Hystad P, Demers PA, Johnson KC, Carpiano RM, Brauer M. Long-term residential exposure to air pollution and lung cancer risk. Epidemiology 2013; 24: 762–72.

Indian Institute of Health Management Research, International Institute of Population Sciences, Ministry of Health and Family Welfare, Government of India, Performance Monitoring and Accountability 2020 (PMA2020) Project, Bill & Melinda Gates Institute for Population and Reproductive Health, Johns Hopkins Bloomberg School of Public Health. PMA 2016 Rajasthan Round 1 Survey Data as part of PMA2020 Project. Baltimore, United States: PMA2020.

International Institute for Population Sciences (IIPS), Ministry of Health and Family Welfare, Government of India, Harvard T. H. Chan School of Public Health, RAND Corporation, Monash University, University of California Los Angeles, Indian Academy of Geriatrics, National AIDS Research Institute, Columbia University. Longitudinal Aging Study in India, Pilot Data 2010. Mumbai, India: IIPS.

International Institute for Population Sciences (IIPS), Ministry of Health and Family Welfare, Government of India, Macro International. India National Family Health Survey Data (NFHS-1) 1992-1993. Mumbai, India: IIPS.

International Institute for Population Sciences (IIPS), Ministry of Health and Family Welfare, Government of India, Macro International. India National Family Health Survey (NFHS-1) 1992-1993: National Report. Mumbai, India: IIPS.

International Institute for Population Sciences (IIPS), Ministry of Health and Family Welfare, Government of India, ORC Macro. India National Family Health Survey Data (NFHS-2) 1998-1999. Mumbai, India: IIPS.

International Institute for Population Sciences (IIPS), Ministry of Health and Family Welfare, Government of India, ORC Macro. India National Family Health Survey (NFHS-2) 1998-1999: National and State Reports. Mumbai, India: IIPS.

International Institute for Population Sciences (IIPS), Ministry of Health and Family Welfare, Government of India, Macro International. India National Family Health Survey Data (NFHS-3) 2005-2006. Mumbai, India: IIPS.

International Institute for Population Sciences (IIPS), Ministry of Health and Family Welfare, Government of India, Macro International. India National Family Health Survey (NFHS-3) 2005-2006: National and State Reports. Mumbai, India: IIPS.

International Institute for Population Sciences (IIPS), Ministry of Health and Family Welfare, Government of India, ICF. India National Family Health Survey data (NFHS-4) 2015-2016. Mumbai, India: IIPS.

International Institute for Population Sciences (IIPS), Ministry of Health and Family Welfare, Government of India, ICF. India National Family Health Survey (NFHS-4) 2015-2016: National and State Reports. Mumbai, India: IIPS.

International Institute for Population Sciences (IIPS), Ministry of Health and Family Welfare, Government of India. India District Level Household Survey Data (DLHS-1) 1998-1999. Mumbai, India: IIPS.

International Institute for Population Sciences (IIPS), Ministry of Health and Family Welfare, Government of India. India District Level Household Survey (DLHS-1) 1998-1999: National Report. Mumbai, India: IIPS.
International Institute for Population Sciences (IIPS), Ministry of Health and Family Welfare, Government of India. India District Level Household Survey Data (DLHS-2) 2002-2004. Mumbai, India: IIPS.
International Institute for Population Sciences (IIPS), Ministry of Health and Family Welfare, Government of India. India District Level Household Survey (DLHS-2) 2002-2004: National and State Reports. Mumbai, India: IIPS.
International Institute for Population Sciences (IIPS), Ministry of Health and Family Welfare, Government of India. India District Level Household and Facility Survey Data (DLHS-3) 2007-2008. Mumbai, India: IIPS.
International Institute for Population Sciences (IIPS), Ministry of Health and Family Welfare, Government of India. India District Level Household and Facility Survey (DLHS-3) 2007-2008: National and State Reports. Mumbai, India: IIPS.
International Institute for Population Sciences, Ministry of Health and Family Welfare, Government of India. India District Level Household and Facility Survey Data (DLHS-4) 2012-2013. Mumbai, India: IIPS.
International Institute for Population Sciences (IIPS), Ministry of Health and Family Welfare, Government of India. India District Level Household and Facility Survey (DLHS-4) 2012-2013: State Reports. Mumbai, India: IIPS.
International Institute for Population Sciences, World Health Organization (WHO). India WHO Study on Global Ageing and Adult Health 2007-2008.
International Institute for Population Sciences, World Health Organization. India World Health Survey 2003.
Jary H, Simpson H, Havens D, et al. Household air pollution and acute lower respiratory infections in adults: a systematic review. Plos One 2016; 11: e0167656.
Jet Propulsion Laboratory, National Aeronautics and Space Administration. Aerosol Optical Depth data from Multi-angle Imaging Spectro Radiometer. Available from: https://www-misr.jpl.nasa.gov/getData/accessData/
Jindal S, Aggarwal A, Chaudhry K, et al. A multicentric study on epidemiology of chronic obstructive pulmonary disease and its relationship with tobacco smoking and environmental tobacco smoke exposure. Indian J Chest Dis Allied Sci 2006; 48:23–29.
Karr C, Lumley T, Schreuder A, et al. Effects of subchronic and chronic exposure to ambient air pollutants on infant bronchiolitis. Am J Epidemiol 2007; 165: 553–60.
Karr CJ, Rudra CB, Miller KA, et al. Infant exposure to fine particulate matter and traffic and risk of hospitalization for RSV bronchiolitis in a region with lower ambient air pollution. Environ Res 2009; 109: 321–7.
Katanoda K, Sobue T, Satoh H, et al. An association between long-term exposure to ambient air pollution and mortality from lung cancer and respiratory diseases in Japan. J Epidemiol 2011; 21: 132–43.
Kim C, Seow WJ, Shu X-O, et al. Cooking coal use and all-cause and cause-specific mortality in a prospective cohort study of women in Shanghai, China. Environ Health Perspect 2016; 124: 1384–9.
Kurmi OP, Arya PH, Lam K-BH, Sorahan T, Ayres JG. Lung cancer risk and solid fuel smoke exposure: a systematic review and meta-analysis. Eur Respir J 2012; 40: 1228–37.
Lepeule J, Laden F, Dockery D, Schwartz J. Chronic exposure to fine particles and mortality: an extended follow-up of the Harvard six cities study from 1974 to 2009. Environ Health Perspect 2012; 120: 965–70.
Lipsett MJ, Ostro BD, Reynolds P, et al. Long-term exposure to air pollution and cardiorespiratory disease in the California teachers study cohort. Am J Respir Crit Care Med 2011; 184: 828–35.
MacIntyre EA., Gehring U, Molter A, et al. Air pollution and respiratory infections during early childhood: an analysis of 10 European birth cohorts within the ESCAPE project. Environ Health Perspect 2014; 122: 107–13.
Mehta S, Shin H, Burnett R, North T, Cohen AJ. Ambient particulate air pollution and acute lower respiratory infections: a systematic review and implications for estimating the global burden of disease. Air Qual Atmos Health 2013; 6: 69–83.
Miller KA, Siscovick DS, Sheppard L, et al. Long-term exposure to air pollution and incidence of cardiovascular events in women. N Engl J Med 2007; 356: 447–58.
Ministry of Health and Family Welfare, Government of India, United Nations Children's Fund. India Coverage Evaluation Survey Data 2009-2010. [Data shared for this analysis]
Ministry of Statistics and Programme Implementation, Government of India. Employment and Unemployment Survey, National Sample Survey Round 38, January-December 1983. New Delhi, India: Ministry of Statistics and Programme Implementation.
Ministry of Statistics and Programme Implementation, Government of India. Survey on Participation in Education, National Sample Survey Round 42, July 1986- June 1987. New Delhi, India: Ministry of Statistics and Programme Implementation.
Ministry of Statistics and Programme Implementation, Government of India. Employment and Unemployment Survey, National Sample Survey Round 43, July 1987- June 1988. New Delhi, India: Ministry of Statistics and Programme Implementation.
Ministry of Statistics and Programme Implementation, Government of India. Survey on Literacy and Culture, National Sample Survey Round 47, July - December 1991. New Delhi, India: Ministry of Statistics and Programme Implementation.

Ministry of Statistics and Programme Implementation, Government of India. Consumer Expenditure Survey, National Sample Survey Round 50, July 1993-June 1994. New Delhi, India: Ministry of Statistics and Programme Implementation.
Ministry of Statistics and Programme Implementation, Government of India. Employment and Unemployment Survey, National Sample Survey Round 50, July 1993- June 1994. New Delhi, India: Ministry of Statistics and Programme Implementation.
Ministry of Statistics and Programme Implementation, Government of India. Consumer Expenditure Survey, National Sample Survey Round 51, July 1994-June 1995. New Delhi, India: Ministry of Statistics and Programme Implementation.
Ministry of Statistics and Programme Implementation, Government of India. Employment and Unemployment Survey, National Sample Survey Round 51, July 1994-June 1995. New Delhi, India: Ministry of Statistics and Programme Implementation.
Ministry of Statistics and Programme Implementation, Government of India. Participation in Education Survey, National Sample Survey Round 52, July 1995-June 1996. New Delhi, India: Ministry of Statistics and Programme Implementation.
Ministry of Statistics and Programme Implementation, Government of India. Survey on Healthcare, National Sample Survey Round 52, July 1995-June 1996. New Delhi, India: Ministry of Statistics and Programme Implementation.
Ministry of Statistics and Programme Implementation, Government of India. Employment and Unemployment Survey, India National Sample Survey Round 55, July 1999-June 2000. New Delhi, India: Ministry of Statistics and Programme Implementation.
Ministry of Statistics and Programme Implementation, Government of India. Consumer Expenditure Survey, National Sample Survey Round 56, July 2000 - June 2001. New Delhi, India: Ministry of Statistics and Programme Implementation.
Ministry of Statistics and Programme Implementation, Government of India. Employment and Unemployment Survey, National Sample Survey Round 61, July 2004- June 2005. New Delhi, India: Ministry of Statistics and Programme Implementation.
Ministry of Statistics and Programme Implementation, Government of India. Consumer Expenditure Survey, National Sample Survey Round 63, July 2006- June 2007. New Delhi, India: Ministry of Statistics and Programme Implementation.
Ministry of Statistics and Programme Implementation, Government of India. Participation & Expenditure in Education Survey, National Sample Survey Round 64, July 2007- June 2008. New Delhi, India: Ministry of Statistics and Programme Implementation.
Ministry of Statistics and Programme Implementation, Government of India. Social Consumption Survey on Health and Education, National Sample Survey Round 71, January 2014 - June 2014. New Delhi, India: Ministry of Statistics and Programme Implementation.
Ministry of Statistics and Programme Implementation, Government of India. Household expenditure on services and durable goods survey, National Sample Survey Round 72, July 2014 - June 2015. New Delhi, India: Ministry of Statistics and Programme Implementation.
Ministry of Statistics and Programme Implementation, Government of India. India State Series: Gross State Domestic Product at Factor Cost by Industry of Origin Tables 1980-81 to 1993-94. New Delhi, India: Ministry of Statistics and Programme Implementation.
Ministry of Statistics and Programme Implementation, Government of India. India State Series: Gross State Domestic Product at Factor Cost by Industry of Origin Tables 1993-94 to 2004-05. New Delhi, India: Ministry of Statistics and Programme Implementation.
Ministry of Statistics and Programme Implementation, Government of India. India State Series: Gross State Domestic Product at Factor Cost by Industry of Origin Tables 1999-2000 to 2009-10. New Delhi, India: Ministry of Statistics and Programme Implementation.
Ministry of Statistics and Programme Implementation, Government of India. India State Series: Gross State Domestic Product at Factor Cost by Industry of Origin Tables 2004-05 to 2013-14. New Delhi, India: Ministry of Statistics and Programme Implementation.
Mishra V, Retherford RD. Cooking smoke increases the risk of acute respiratory infection in children. Nat Fam Health Surv Bull. 1997; 1–4.
Mishra V, Smith KR, Retherford RD. Effects of cooking smoke and environmental tobacco smoke on acute respiratory infections in young Indian children. Pop Environ. 2005; 26: 375–396.
Mitter SS, Vedanthan R, Islami F, et al. Household fuel use and cardiovascular disease mortality: Golestan cohort study. Circulation 2016; 133: 2360–9.
Mohan M, Sperduto RD, Angra SK, Milton RC, Mathur RL, Underwood BA, et al. India–US case-control study of age-related cataracts. India–US Case-Control Study Group. Arch Ophthalmol 1989; 107: 670–676.
Mukherjee B, Dutta A, Roychoudhury S, Ray MR. Chronic inhalation of biomass smoke is associated with DNA damage in airway cells: involvement of particulate pollutants and benzene. J Appl Toxicol 2013; 33: 281-289.
Næss Ø, Nafstad P, Aamodt G, Claussen B, Rosland P. Relation between concentration of air pollution and cause-specific mortality: four-year exposures to nitrogen dioxide and particulate matter pollutants in 470 neighbourhoods in Oslo, Norway. Am J Epidemiol 2007; 165: 435–43.

National Aeronautics and Space Administration (NASA), Center for International Earth Science Information Network, Columbia University. Gridded Population of the World, Version 4 (GPWv4): Population Count Adjusted to Match 2015 Revision of UN WPP Country Totals. Palisades, United States: NASA SEDAC; 2016. Available from: http://sedac.ciesin.columbia.edu/data/set/gpw-v4-population-count-adjusted-to-2015-unwpp-country-totals.

National Aeronautics and Space Administration. Aerosol Optical Depth data from Aerosol Robotic Network. Available from: https://aeronet.gsfc.nasa.gov/new_web/data.html

National Aeronautics and Space Administration. Aerosol Optical Depth data from Moderate Resolution Imaging Spectroradiometer- Dark Target Aerosol Retrieval Algorithm. Available from: https://darktarget.gsfc.nasa.gov/algorithm

National Aeronautics and Space Administration. Aerosol Optical Depth data from Moderate Resolution Imaging Spectroradiometer- Multi-Angle Implementation of Atmospheric Correction. Available from: https://ladsweb.modaps.eosdis.nasa.gov/api/v1/productGroupPage/name=maiac

National Aeronautics and Space Administration. Aerosol Optical Depth data from Moderate Resolution Imaging Spectroradiometer. Available from: https://modis.gsfc.nasa.gov/data/

National Aeronautics and Space Administration. SeaWiFS Deep Blue Aerosol Optical Depth and Angstrom Exponent Monthly Level 3 Data Gridded at 1.0 Degrees. Available from: https://data.nasa.gov/Earth-Science/SeaWiFS-Deep-Blue-Aerosol-Optical-Depth-and-Angstr/h7mm-kenx

Newby DE, Mannucci PM, Tell GS, et al. Expert position paper on air pollution and cardiovascular disease. Eur Heart J 2015; 36: 83–93b.

Office of the Registrar General & Census Commissioner, Ministry of Home Affairs, Government of India. India Annual Health Survey Data 2010-2011. New Delhi, India: Office of the Registrar General & Census Commissioner.

Office of the Registrar General & Census Commissioner, Ministry of Home Affairs, Government of India. India Annual Health Survey Data 2011-2012. New Delhi, India: Office of the Registrar General & Census Commissioner.

Office of the Registrar General & Census Commissioner, Ministry of Home Affairs, Government of India. India Annual Health Survey Data 2012-2013. New Delhi, India: Office of the Registrar General & Census Commissioner.

Office of the Registrar General & Census Commissioner, Ministry of Home Affairs, Government of India. India Population and Housing Census 2011. New Delhi, India: Office of the Registrar General & Census Commissioner; 2012.

Parikha J, Balakrishnanb K, Laxmia V, Biswasa H. Exposure from cooking with biofuels: pollution monitoring and analysis for rural Tamil Nadu, India. Energy. 2001; 26: 949–62.

Park SK, Adar SD, O'Neill MS, et al. Long-term exposure to air pollution and type 2 diabetes mellitus in a multiethnic cohort. Am J Epidemiol 2015; 181: 327–36.

Parker JD, Kravets N, Vaidyanathan A. Particulate matter air pollution exposure and heart disease mortality risks by race and ethnicity in the United States. Circulation 2018; 137: 1688–97.

Pinault L, Tjepkema M, Crouse DL, et al. Risk estimates of mortality attributed to low concentrations of ambient fine particulate matter in the Canadian community health survey cohort. Environ Health 2016; 15: 18.

Puett RC, Hart JE, Schwartz J, Hu FB, Liese AD, Laden F. Are particulate matter exposures associated with risk of type 2 diabetes? Environ Health Perspect 2011; 119: 384–9.

Puett RC, Hart JE, Yanosky JD, et al. Chronic fine and coarse particulate exposure, mortality, and coronary heart disease in the Nurses' Health Study. Environ Health Perspect 2009; 117: 1697–701.

Qureshi K. Domestic smoke pollution and prevalence of chronic bronchitis/asthma in a rural area of Kashmir. Indian J Chest Diseases Allied Sci 1994; 36: 61–72.

Raaschou-Nielsen O, Andersen ZJ, Beelen R, et al. Air pollution and lung cancer incidence in 17 European cohorts: prospective analyses from the European Study of Cohorts for Air Pollution Effects (ESCAPE). Lancet Oncol 2013; 14: 813–22.

Raaschou-Nielsen O, Beelen R, Wang M, et al. Particulate matter air pollution components and risk for lung cancer. Environ Int 2016; 87: 66–73.

Saha A, Kulkarni P, Shah A, Patel M, Saiyed H. Ocular morbidity and fuel use: An experience from India. Occup Environ Med 2005; 62:66–69.

Sapkota A, Gajalakshmi V, Jetly D, Roychowdhury S, Dikshit R. Indoor air pollution from solid fuels and risk of hypopharyngeal/laryngeal and lung cancers: A multicentric case-control study from India. Int J Epidemiol 2008; 37: 321–328.

Scheers H, Jacobs L, Casas L, Nemery B, Nawrot TS. Long-term exposure to particulate matter air pollution is a risk factor for stroke: meta-analytical evidence. Stroke 2015; 46: 3058–66.

Schultz MG, Schröder S, Lyapina O, et al. Tropospheric Ozone Assessment Report: database and metrics data of global surface ozone observations. Elem Sci Anth 2017; 5: 58.

Smith KR, Bruce N, Balakrishnan K, et al. Millions dead: how do we know and what does it mean? Methods used in the comparative risk assessment of household air pollution. Annu Rev Public Health 2014; 35: 185–206.

Smith KR, McCracken JP, Weber MW, et al. Effect of reduction in household air pollution on childhood pneumonia in Guatemala (RESPIRE): a randomised controlled trial. Lancet 2011; 378: 1717–26.

Sreenivas V, Prabhakar A, Badrinath S, Fernandez T, Roy I, Sharma T, et al. A rural population based case-control study of senile cataract in India. J Epidemiol 1999; 9: 327–336.
Thurston GD, Ahn J, Cromar KR, et al. Ambient particulate matter air pollution exposure and mortality in the NIH-AARP diet and health cohort. Environ Health Perspect 2016; 124: 484–90.
Tiwari S, Hopke PK, Pipal AS, Srivastava AK, Bishta DS, Tiwari S, Singh AK, Sonie VK, Attrie SD. Intra-urban variability of particulate matter (PM2.5 and PM10) and its relationship with optical properties of aerosols over Delhi, India. Atmos Res. 2015; 166: 223-32.
To T, Zhu J, Villeneuve PJ, et al. Chronic disease prevalence in women and air pollution: a 30-year longitudinal cohort study. Environ Int 2015; 80: 26–32.
Tseng E, Ho W-C, Lin M-H, Cheng T-J, Chen P-C, Lin H-H. Chronic exposure to particulate matter and risk of cardiovascular mortality: cohort study from Taiwan. BMC Public Health 2015; 15: 936.
Turner MC, Jerrett M, Pope CA, et al. Long-term ozone exposure and mortality in a large prospective study. Am J Respir Crit Care Med 2016; 193: 1134–42.
Villeneuve PJ, Weichenthal SA, Crouse D, et al. Long-term exposure to fine particulate matter air pollution and mortality among Canadian women. Epidemiology 2015; 26: 536–45.
Weichenthal S, Hoppin JA, Reeves F. Obesity and cardiovascular health effects of fine particulate air pollution. Obesity (Silver Spring) 2014; 22: 1580–9.
Weichenthal S, Pinault LL, Burnett RT. Impact of oxidant gases on the relationship between outdoor fine particulate air pollution and nonaccidental, cardiovascular, and respiratory mortality. Sci Rep 2017; 7: 16401.
Weinmayr G, Hennig F, Fuks K, et al. Long-term exposure to fine particulate matter and incidence of type 2 diabetes mellitus in a cohort study: effects of total and traffic-specific air pollution. Environ Health 2015; 14: 53.
West S, Bates M, Lee J, et al. Is household air pollution a risk factor for eye disease? Int J Environ Res Public Health 2013; 10, 5378–98.
Wong CM, Lai HK, Tsang H, et al. Satellite-based estimates of long-term exposure to fine particles and association with mortality in elderly Hong Kong residents. Environ Health Perspect 2015; 123: 1167–72.
World Bank. Survey of Living Conditions 1997-1998, Uttar Pradesh and Bihar. Washington D.C., United States: World Bank.
World Health Organization (WHO). WHO Household Energy Database. Geneva, Switzerland: WHO; 2010.
World Health Organization (WHO). WHO Urban Ambient Air Pollution Database Draft 2016. Geneva, Switzerland: WHO.
Yin P, Brauer M, Cohen A, et al. Long-term fine particulate matter exposure and nonaccidental and cause-specific mortality in a large national cohort of Chinese men. Environ Health Perspect 2017; 125: 117002.
Yu K, Qiu G, Chan K-H, et al. Association of solid fuel use with risk of cardiovascular and all-cause mortality in rural China. JAMA 2018; 319: 1351–61.
Zodpey SP, Ughade SN. Exposure to cheaper cooking fuels and risk of age-related cataract in women. Indian J Occup Environ Med. 1999; 3:159–161.

4. Grouping of the states of India based on Socio-demographic Index, 2017

State group (population in 2017)	States of India	SDI in 2017
**Low SDI states (675 million)**	Bihar	0.43
	Madhya Pradesh	0.49
	Jharkhand	0.49
	Uttar Pradesh	0.49
	Rajasthan	0.49
	Chhattisgarh	0.51
	Odisha	0.52
	Assam	0.53
**Middle SDI states (387 million)**	Andhra Pradesh	0.54
	West Bengal	0.54
	Tripura	0.54
	Arunachal Pradesh	0.56
	Meghalaya	0.56
	Karnataka	0.57
	Telangana	0.58
	Gujarat	0.58
	Manipur	0.59
	Jammu and Kashmir	0.59
	Haryana	0.60
**High SDI states (318 million)**	Uttarakhand	0.61
	Tamil Nadu	0.62
	Mizoram	0.62
	Maharashtra	0.62
	Punjab	0.62
	Sikkim	0.63
	Nagaland	0.63
	Himachal Pradesh	0.63
	Union territories other than Delhi	0.65
	Kerala	0.66
	Delhi	0.72
	Goa	0.74

SDI calculated by GBD as described elsewhere (Lancet 2018; 392: 1995-2051).
SDI=Socio-demographic Index.

5. Top ten countries with the highest exposure level to ambient particulate matter pollution, 2017

Countries	Population-weighted annual mean $PM_{2.5}$ μg/m^3 (95% uncertainty interval)
Nepal	99.7 (44.6 to 179.5)
Niger	94.1 (18.6 to 306.8)
Qatar	91.2 (55.5 to 140.7)
India	89.8 (66.9 to 111.8)
Saudi Arabia	87.9 (29.6 to 197.9)
Egypt	87.0 (33.7 to 164.2)
Cameroon	72.8 (22.2 to 167.7)
Nigeria	71.8 (18.8 to 270.6)
Bahrain	70.8 (49.1 to 99.4)
Chad	66.0 (14.9 to 194.0)

Source: Global Burden of Diseases, Injuries, and Risk Factors Study 2017

6. Mean $PM_{2.5}$ level and proportion of population using solid fuels in each state of India, 2017

States of India	Population-weighted mean $PM_{2.5}$ µg/m^3 (95% uncertainty interval)	Percentage of population using solid fuels (95% uncertainty interval)
**India**	**89.9 (67.0 to 112.0)**	**55.5 (54.8 to 56.2)**
**Low SDI states**	**125.3 (87.5 to 167.3)**	**72.1 (71.1 to 73.0)**
Bihar	169.4 (83.9 to 266.5)	81.5 (79.1 to 83.7)
Madhya Pradesh	77.9 (47.0 to 125.5)	69.8 (67.6 to 71.6)
Jharkhand	86.8 (53.6 to 147.1)	79.1 (77.1 to 81.1)
Uttar Pradesh	174.7 (101.3 to 276.9)	66.9 (64.3 to 69.1)
Rajasthan	93.4 (55.7 to 144.4)	68.0 (65.9 to 70.0)
Chhattisgarh	52.5 (34.3 to 73.2)	74.8 (72.6 to 77.0)
Odisha	49.2 (31.3 to 70.5)	76.7 (74.1 to 78.9)
Assam	40.2 (28.5 to 53.7)	73.4 (70.7 to 75.9)
**Middle SDI states**	**58.7 (44.8 to 76.6)**	**46.7 (45.7 to 47.8)**
Andhra Pradesh	39.0 (29.5 to 49.6)	30.7 (28.1 to 33.7)
West Bengal	81.4 (56.7 to 107.5)	66.4 (63.9 to 68.5)
Tripura	46.4 (34.3 to 61.8)	61.0 (57.7 to 64.0)
Arunachal Pradesh	27.1 (17.5 to 37.9)	55.4 (52.1 to 58.4)
Meghalaya	36.6 (27.9 to 49.0)	73.6 (70.8 to 76.1)
Karnataka	32.2 (18.2 to 49.9)	42.8 (40.4 to 45.2)
Telangana	47.2 (34.5 to 66.0)	28.1 (24.2 to 32.1)
Gujarat	49.4 (26.5 to 84.7)	43.4 (41.1 to 45.8)
Manipur	42.9 (30.6 to 58.4)	58.3 (55.2 to 61.4)
Jammu and Kashmir	57 (35.4 to 86.5)	41.8 (38.8 to 44.8)
Haryana	125.7 (65.2 to 194.5)	45.7 (43.2 to 48.1)
**High SDI states**	**56.6 (44.0 to 71.6)**	**31.0 (30.0 to 32.1)**
Uttarakhand	73.4 (38.4 to 122.3)	46.0 (43.0 to 48.9)
Tamil Nadu	32.1 (18.2 to 61.4)	21.0 (19.1 to 22.9)
Mizoram	32.8 (25.3 to 42.4)	34.2 (31.7 to 36.5)
Maharashtra	55.7 (38.5 to 72.7)	37.7 (35.6 to 39.7)
Punjab	79.6 (50.4 to 116.9)	31.9 (29.5 to 34.2)
Sikkim	50.3 (35.2 to 71.8)	34.8 (32.0 to 37.8)
Nagaland	40.3 (31.1 to 51.7)	64.1 (61.1 to 67.0)
Himachal Pradesh	38.6 (23.3 to 58.5)	58.1 (54.3 to 61.5)
Union territories other than Delhi	47.8 (22.3 to 95.7)	14.9 (13.2 to 17.1)
Kerala	17.3 (14.2 to 21.2)	35.5 (32.5 to 38.3)
Delhi	209.0 (120.9 to 339.5)	1.9 (1.3 to 2.7)
Goa	24.5 (18.2 to 39.2)	10.5 (8.7 to 12.4)

SDI=Socio-demographic Index.

7. Deaths attributable to ambient particulate matter and household air pollution by sex in the states of India, 2017

States of India	Number of deaths attributable to ambient particulate matter pollution (95% uncertainty interval)		Number of deaths attributable to household air pollution (95% uncertainty interval)	
	Females	Males	Females	Males
**India**	**282,529 (223,685 to 338,895)**	**390,600 (321,693 to 462,962)**	**260,269 (209,821 to 314,080)**	**221,469 (172,140 to 281,325)**
**Low SDI states**	**144,879 (108,433 to 182,647)**	**195,312 (151,243 to 242,478)**	**139,139 (108,593 to 172,216)**	**119,148 (89,991 to 154,677)**
Bihar	24,556 (15,107 to 33,619)	29,077 (18,324 to 39,226)	21,447 (14,430 to 30,001)	16,378 (10,509 to 24,246)
Madhya Pradesh	16,345 (11,290 to 23,366)	21,400 (15,065 to 29,295)	21, 187 (15,114 to 27,730)	18,708 (12,856 to 24,908)
Jharkhand	5,607 (3,880 to 7,931)	6,446 (4,671 to 8,746)	7,789 (5,665 to 9,960)	4,979 (3,429 to 6,633)
Uttar Pradesh	68,763 (44,952 to 94,277)	92,416 (65,298 to 123,182)	43, 002 (27,013 to 62,232)	35,886 (21,136 to 53,954)
Rajasthan	17,021 (10,172 to 24,719)	26,274 (17,358 to 35,899)	20,088 (13,032 to 27,368)	19,201 (12,968 to 26,543)
Chhattisgarh	4,115 (2,770 to 5,722)	7,028 (4,957 to 9,221)	8,702 (6,849 to 10,700)	8,326 (6,150 to 10,677)
Odisha	4,571 (2,927 to 6,926)	7,414 (4,939 to 10,321)	8,819 (6,628 to 11, 841)	8,815 (6,564 to 11,596)
Assam	3,899 (2,768 to 5,241)	5,257 (3,799 to 7,060)	8,105 (6,423 to 9,996)	6,857 (5,181 to 8,799)
**Middle SDI states**	**70,808 (55,880 to 87,638)**	**102,593 (82,857 to 124,138)**	**74,263 (59,306 to 90,084)**	**64,790 (49,175 to 81,124)**
Andhra Pradesh	9,446 (6,618 to 13,155)	13,835 (10,181 to 18,232)	10, 494 (7,253 to 14, 329)	8,852 (5,804 to 12,263)
West Bengal	20,677 (15,182 to 26,568)	29,205 (22,190 to 36,453)	21,203 (15,797 to 27,167)	17,643 (12,415 to 23,748)
Tripura	622 (455 to 820)	1,005 (762 to 1,263)	945 (704 to 1,229)	898 (672 to 1,138)
Arunachal Pradesh	71.1 (43.4 to 104)	126 (81 to 179)	185 (140 to 245)	178 (125 to 237)
Meghalaya	211 (152 to 292)	309 (223 to 412)	428 (313 to 551)	419 (309 to 546)
Karnataka	10,838 (7,036 to 15,481)	15,473 (10,295 to 21,646)	17,716 (13,149 to 22,501)	15,981 (11,552 to 20,778)
Telangana	6,083 (4,381 to 8,198)	9,156 (6,909 to 12,194)	4,697 (3,174 to 6,635)	4,091 (2,663 to 5,814)
Gujarat	12,360 (8,087 to 17,672)	17,431 (11,898 to 24,161)	12,729 (8,872 to 16,545)	11,440 (7,718 to 15,414)
Manipur	388 (275 to 546)	556 (400 to 741)	481 (355 to 653)	427 (295 to 571)
Jammu and Kashmir	2,385 (1,624 to 3,263)	3,437 (2,493 to 4,507)	1,889 (1,331 to 2,528)	1,607 (1,055 to 2,280)
Haryana	7,727 (5,298 to 10,280)	12,060 (8,730 to 15,317)	3,495 (2,214 to 5,147)	3,255 (1,935 to 5,178)
**High SDI states**	**66,843 (53,926 to 81,008)**	**92,695 (76,488 to 110,492)**	**46,867 (37,018 to 57,311)**	**37,531 (28,476 to 48,183)**
Uttarakhand	2,666 (1,627 to 3,814)	4,293 (2,870 to 5,818)	1,811 (1,170 to 2,576)	1,759 (1,064 to 2,596)
Tamil Nadu	15,877 (10,883 to 22,173)	23,983 (17,352 to 32,241)	10, 305 (7,367 to 13,371)	9,320 (6,194 to 12,877)
Mizoram	149 (104 to 200)	191 (138 to 246)	131 (95 to 175)	112 (76 to 149)
Maharashtra	28,011 (20,517 to 35,848)	34,667 (26,899 to 42,869)	22,247 (15,982 to 28,758)	14,684 (10,071 to 20,066)
Punjab	7,944 (6,020 to 9,921)	11,234 (8,932 to 13,756)	3,396 (2,336 to 4, 704)	2,744 (1,696 to 3,970)
Sikkim	91 (60 to 126)	152 (107 to 196)	67.5 (44.9 to 93.6)	64 (41 to 92)
Nagaland	169 (122 to 231)	259 (192 to 340)	253 (183 to 347)	240 (167 to 329)
Himachal Pradesh	1,051 (620 to 1,539)	2,256 (1,441 to 3,184)	1,300 (916 to 1, 735)	1,686 (1,111 to 2,426)
Union territories other than Delhi	498 (320 to 710)	864 (563 to 1,255)	175 (117 to 246)	166 (106 to 244)
Kerala	4,870 (3,698 to 6,341)	7,884 (6,111 to 10,198)	7,078 (5,447 to 8,740)	6,680 (4,916 to 8,741)
Delhi	5,219 (4,160 to 6,356)	6,512 (5,238 to 7,850)	30 (15 to 54)	22 (11 to 41)
Goa	298 (228 to 396)	402 (306 to 527)	73 (49 to 104)	57 (34 to 84)

SDI=Socio-demographic Index.

8. Impact of air pollution on life expectancy in the states of India by sex, 2017

States of India*	Life expectancy at birth in years in 2017 (95% uncertainty interval)		Increase in life expectancy in years if air pollution levels were less than the minimum level causing health loss (95% uncertainty interval)					
			Ambient particulate matter pollution		Household air pollution		Air pollution	
	Female	Male	Female	Male	Female	Male	Female	Male
**India**	**70.2 (69.6 to 70.7)**	**67.8 (67.4 to 68.3)**	**0.8 (0.7 to 1.0)**	**1.0 (0.9 to 1.2)**	**0.7 (0.6 to 0.9)**	**0.6 (0.5 to 0.7)**	**1.7 (1.5 to 1.9)**	**1.8 (1.6 to 2.0)**
Bihar	69.0 (68.0 to 70.0)	70.2 (69.2 to 71.1)	1.0 (0.6 to 1.3)	1.1 (0.8 to 1.4)	0.8 (0.6 to 1.1)	0.6 (0.4 to 0.8)	2.0 (1.7 to 2.2)	1.9 (1.6 to 2.2)
Madhya Pradesh	68.4 (67.5 to 69.5)	65.8 (64.8 to 66.8)	0.7 (0.5 to 0.9)	0.9 (0.7 to 1.2)	0.9 (0.7 to 1.1)	0.8 (0.6 to 1.1)	1.7 (1.5 to 1.9)	2.0 (1.7 to 2.2)
Jharkhand	68.0 (67.1 to 69.0)	69.2 (68.3 to 70.0)	0.6 (0.5 to 0.9)	0.8 (0.6 to 1.0)	0.9 (0.7 to 1.1)	0.6 (0.5 to 0.8)	1.7 (1.5 to 1.9)	1.6 (1.4 to 1.8)
Uttar Pradesh	66.1 (64.9 to 67.2)	65.1 (64.0 to 66.2)	1.2 (0.8 to 1.5)	1.4 (1.1 to 1.8)	0.7 (0.5 to 0.9)	0.5 (0.4 to 0.8)	2.1 (1.7 to 2.5)	2.2 (1.8 to 2.6)
Rajasthan	70.6 (69.6 to 71.6)	65.9 (64.8 to 67.0)	0.9 (0.6 to 1.3)	1.3 (0.9 to 1.6)	1.1 (0.8 to 1.4)	0.9 (0.7 to 1.2)	2.3 (1.8 to 2.7)	2.5 (2.2 to 2.9)
Chhattisgarh	67.0 (66.1 to 68.0)	62.1 (61.2 to 63.0)	0.5 (0.3 to 0.6)	0.6 (0.5 to 0.8)	1.0 (0.8 to 1.2)	0.8 (0.6 to 1.0)	1.6 (1.4 to 1.8)	1.5 (1.4 to 1.7)
Odisha	69.8 (69.0 to 70.7)	67.2 (66.3 to 68.1)	0.4 (0.3 to 0.5)	0.5 (0.4 to 0.7)	0.7 (0.6 to 0.9)	0.6 (0.5 to 0.8)	1.2 (1.0 to 1.5)	1.2 (1.0 to 1.4)
Assam	68.2 (67.2 to 69.1)	65.5 (64.6 to 66.5)	0.4 (0.3 to 0.6)	0.6 (0.4 to 0.7)	0.9 (0.8 to 1.1)	0.8 (0.6 to 0.9)	1.5 (1.3 to 1.7)	1.4 (1.3 to 1.6)
Andhra Pradesh	72.4 (70.1 to 74.4)	69.5 (67.3 to 71.6)	0.5 (0.4 to 0.7)	0.8 (0.7 to 1.0)	0.6 (0.5 to 0.7)	0.5 (0.4 to 0.7)	1.2 (1.1 to 1.4)	1.5 (1.3 to 1.7)
West Bengal	72.2 (71.1 to 73.2)	69.8 (68.7 to 70.9)	0.8 (0.6 to 1.0)	1.0 (0.8 to 1.1)	0.8 (0.6 to 1.0)	0.6 (0.5 to 0.8)	1.8 (1.6 to 2.0)	1.7 (1.5 to 1.9)
Tripura	73.0 (71.3 to 74.3)	67.2 (65.9 to 68.4)	0.6 (0.5 to 0.8)	0.8 (0.7 to 1.0)	0.9 (0.8 to 1.1)	0.7 (0.6 to 0.9)	1.7 (1.5 to 1.9)	1.7 (1.5 to 1.9)
Arunachal Pradesh	73.5 (71.7 to 75.0)	68.5 (67.2 to 70.2)	0.3 (0.2 to 0.4)	0.4 (0.3 to 0.5)	0.8 (0.6 to 0.9)	0.5 (0.4 to 0.7)	1.1 (1.0 to 1.3)	1.0 (0.9 to 1.2)
Meghalaya	73.1 (71.5 to 74.7)	66.8 (65.1 to 68.4)	0.4 (0.3 to 0.5)	0.4 (0.3 to 0.6)	0.8 (0.6 to 0.9)	0.6 (0.5 to 0.7)	1.2 (1.1 to 1.5)	1.1 (1.0 to 1.3)
Karnataka	69.3 (68.4 to 70.1)	66.3 (65.3 to 67.1)	0.5 (0.3 to 0.6)	0.6 (0.4 to 0.8)	0.8 (0.6 to 0.9)	0.7 (0.5 to 0.8)	1.4 (1.2 to 1.5)	1.4 (1.2 to 1.6)
Telangana	73.0 (71.0 to 75.0)	70.0 (68.0 to 71.9)	0.6 (0.5 to 0.7)	0.9 (0.7 to 1.1)	0.5 (0.3 to 0.6)	0.4 (0.3 to 0.5)	1.2 (1.0 to 1.3)	1.4 (1.3 to 1.6)
Gujarat	72.2 (71.2 to 73.1)	68.6 (67.5 to 69.6)	0.7 (0.5 to 0.9)	0.9 (0.7 to 1.2)	0.7 (0.5 to 0.8)	0.6 (0.5 to 0.8)	1.5 (1.3 to 1.8)	1.8 (1.5 to 2.0)
Manipur	73.7 (71.9 to 75.2)	68.2 (66.8 to 69.7)	0.5 (0.4 to 0.7)	0.6 (0.5 to 0.7)	0.6 (0.5 to 0.8)	0.5 (0.3 to 0.6)	1.3 (1.1 to 1.4)	1.1 (1.0 to 1.2)
Jammu and Kashmir	73.6 (72.6 to 74.6)	72.1 (71.0 to 73.1)	1.0 (0.7 to 1.2)	1.2 (0.9 to 1.5)	0.8 (0.6 to 1.0)	0.5 (0.4 to 0.7)	2.0 (1.7 to 2.3)	2.0 (1.7 to 2.2)
Haryana	71.0 (70.1 to 72.0)	67.6 (66.6 to 68.6)	1.2 (0.9 to 1.5)	1.6 (1.2 to 2.0)	0.5 (0.4 to 0.7)	0.4 (0.3 to 0.6)	1.9 (1.6 to 2.2)	2.3 (2.0 to 2.6)
Uttarakhand	73.3 (72.3 to 74.2)	66.7 (65.7 to 67.6)	0.9 (0.6 to 1.2)	1.2 (0.8 to 1.5)	0.6 (0.4 to 0.8)	0.5 (0.3 to 0.7)	1.8 (1.5 to 2.1)	1.9 (1.6 to 2.2)
Tamil Nadu	72.3 (71.5 to 73.1)	68.9 (68.0 to 69.7)	0.6 (0.4 to 0.8)	0.8 (0.6 to 1.1)	0.4 (0.3 to 0.5)	0.3 (0.2 to 0.4)	1.0 (0.9 to 1.2)	1.2 (1.0 to 1.4)
Mizoram	73.5 (71.8 to 75.2)	67.7 (66.3 to 69.4)	0.6 (0.5 to 0.8)	0.6 (0.5 to 0.7)	0.6 (0.4 to 0.7)	0.4 (0.3 to 0.5)	1.4 (1.2 to 1.6)	1.1 (1.0 to 1.3)
Maharashtra	72.7 (71.9 to 73.4)	70.7 (69.8 to 71.5)	0.7 (0.6 to 0.9)	1.0 (0.8 to 1.2)	0.6 (0.4 to 0.7)	0.4 (0.3 to 0.5)	1.5 (1.3 to 1.7)	1.6 (1.4 to 1.7)
Punjab	73.7 (72.8 to 74.6)	71.1 (70.1 to 72.1)	1.1 (0.9 to 1.3)	1.4 (1.2 to 1.7)	0.5 (0.3 to 0.6)	0.3 (0.2 to 0.5)	1.7 (1.5 to 1.9)	1.9 (1.7 to 2.1)
Sikkim	75.5 (74.0 to 77.2)	70.0 (68.7 to 71.8)	0.7 (0.6 to 0.9)	0.9 (0.7 to 1.0)	0.5 (0.4 to 0.7)	0.4 (0.3 to 0.5)	1.4 (1.2 to 1.6)	1.4 (1.2 to 1.5)
Nagaland	73.9 (72.2 to 75.7)	68.2 (66.8 to 70.0)	0.5 (0.4 to 0.6)	0.5 (0.4 to 0.7)	0.7 (0.6 to 0.9)	0.5 (0.4 to 0.6)	1.3 (1.2 to 1.5)	1.1 (1.0 to 1.3)
Himachal Pradesh	76.8 (75.8 to 77.8)	68.5 (67.3 to 69.6)	0.6 (0.4 to 0.8)	0.8 (0.6 to 1.1)	0.7 (0.5 to 0.9)	0.6 (0.5 to 0.8)	1.6 (1.3 to 1.8)	1.8 (1.5 to 2.0)
Union territories other than Delhi	76.1 (74.6 to 77.7)	70.6 (68.8 to 72.4)	0.7 (0.5 to 0.9)	0.9 (0.7 to 1.1)	0.2 (0.2 to 0.3)	0.2 (0.1 to 0.2)	0.9 (0.8 to 1.1)	1.1 (0.9 to 1.4)
Kerala	77.4 (76.6 to 78.3)	71.8 (70.7 to 72.8)	0.3 (0.3 to 0.4)	0.5 (0.4 to 0.6)	0.5 (0.4 to 0.6)	0.4 (0.3 to 0.5)	0.9 (0.8 to 1.0)	1.0 (0.9 to 1.1)
Delhi	75.0 (73.8 to 76.2)	72.4 (71.1 to 73.6)	1.4 (1.2 to 1.6)	1.6 (1.4 to 1.8)	0.0 (0.0 to 0.0)	0.0 (0.0 to 0.0)	1.5 (1.3 to 1.7)	1.7 (1.5 to 1.9)
Goa	78.2 (76.6 to 79.5)	72.5 (70.6 to 74.2)	0.7 (0.6 to 0.8)	0.8 (0.7 to 0.9)	0.2 (0.1 to 0.2)	0.1 (0.1 to 0.1)	0.9 (0.8 to 1.0)	1.0 (0.9 to 1.1)

*States are listed in increasing order of Socio-demographic Index in 2017.
